# WDR1 is a novel EYA3 substrate and its dephosphorylation induces modifications of the cellular actin cytoskeleton

**DOI:** 10.1038/s41598-018-21155-w

**Published:** 2018-02-13

**Authors:** Mihaela Mentel, Aura E. Ionescu, Ioana Puscalau-Girtu, Martin S. Helm, Rodica A. Badea, Silvio O. Rizzoli, Stefan E. Szedlacsek

**Affiliations:** 1 Department of Enzymology, Institute of Biochemistry of the Romanian Academy, Spl. Independentei 296, Bucharest, 060031 Romania; 2 Department for Neuro- and Sensory Physiology, University Medical Center Göttingen, and Center for Nanoscale Microscopy and Molecular Physiology of the Brain, Cluster of Excellence 171, Humboldtalle 23, Göttingen, 37073 Germany; 3 Max-Planck Research School Molecular Biology, Göttingen, 37077 Germany

## Abstract

Eyes absent (EYA) proteins are unusual proteins combining in a single polypeptide chain transactivation, threonine phosphatase, and tyrosine phosphatase activities. They play pivotal roles in organogenesis and are involved in a variety of physiological and pathological processes including innate immunity, DNA damage repair or cancer metastasis. The molecular targets of EYA tyrosine phosphatase activity are still elusive. Therefore, we sought to identify novel EYA substrates and also to obtain further insight into the tyrosine-dephosphorylating role of EYA proteins in various cellular processes. We show here that Src kinase phosphorylates tyrosine residues in two human EYA family members, EYA1 and EYA3. Both can autodephosphorylate these residues and their nuclear and cytoskeletal localization seems to be controlled by Src phosphorylation. Next, using a microarray of phosphotyrosine-containing peptides, we identified a phosphopeptide derived from WD-repeat-containing protein 1 (WDR1) that is dephosphorylated by EYA3. We further demonstrated that several tyrosine residues on WDR1 are phosphorylated by Src kinase, and are efficiently dephosphorylated by EYA3, but not by EYA1. The lack of phosphorylation generates major changes to the cellular actin cytoskeleton. We, therefore, conclude that WDR1 is an EYA3-specific substrate, which implies that EYA3 is a key modulator of the cytoskeletal reorganization.

## Introduction

Eyes absent genes are members of retinal determination network known to be involved in cell-fate determination in various organisms. In vertebrates, this network is composed of gene families *Pax*, *Six*, *Eya* and *Dach* which play essential regulatory roles in the development of eye, heart, ears, muscle, lungs and other organs^1^. While the proteins encoded by *Pax*, *Six* and *Dach* are transcription factors, the EYA proteins encoded by *Eya* genes are unusual by combining in a single polypeptide chain individual domains for transcriptional activation, a threonine phosphatase and a tyrosine phosphatase (PTP), respectively. Both the transcriptional activation and the threonine phosphatase activity are located in the poorly conserved N-terminal domain (NTD)^2,3^, whereas the PTP activity is linked to the highly conserved C-terminal domain (also called EYA domain, ED)^4–6^. There are four vertebrate paralogs of EYA (EYA1-4)^7^. An additional particularity of EYA proteins is that the ED does not belong to the classical PTPs, but to the phosphatase subgroup of haloacid dehalogenases (HAD)^4–6^.

Despite the diversity of the EYA functions, there are only two validated substrates of EYA tyrosine phosphatase activity^8^. The first is H2AX, a histone protein involved in DNA damage repair. It was reported that EYA1, EYA2 and EYA3 are able to dephosphorylate the C-terminal phosphotyrosine 142 (pY142) of H2AX. Phosphorylation of Y142 in H2AX is controlled by tyrosine kinase WSTF^9^ and by the EYA tyrosine phosphatase activity. Thus, the C-terminal pY142 is an essential regulator of the DNA damage response by controlling the recruitment of either DNA repair or pro-apoptotic factors involved in DNA damage repair and signaling^10,11^. The second validated substrate is the β-form of the estrogen receptor (ERβ)^12^. The N-terminal tyrosine Y36 in ERβ can be phosphorylated by c-Abl tyrosine kinase and dephosphorylated by EYA2. Moreover, a correlation has been found between a higher level of Y36 phosphorylation and the ERβ-specific activation of transcription on the one hand and the ERβ-dependent inhibition of tumor cell growth on the other hand.

EYA proteins are intrinsically cytoplasmic, but can be translocated from cytoplasm to nucleus by interacting with SIX proteins^13^. Thus, EYA proteins have distinct functions: in nucleus they are involved in DNA damage repair^10^ and regulation in transcription^4^, in cytoplasm they appear to play a role in cell polarity, cell motility^14,15^ and innate immunity^3^. The EYA substrates - H2AX and ERβ – illustrate the role of EYA as a nuclear protein. Although the cytoplasmic role of EYA has been clearly demonstrated^16^ there are very limited data regarding the cytoplasmic substrates of any of EYA family members. An indication concerning the cytoplasmic substrates of EYA family of proteins is that EYA of *Drosophila* is able to use its own phosphorylated form as a substrate^6^. However, it is still unclear whether human EYA proteins are able to autodephosphorylate. In addition, it is unknown which kinase can phosphorylate tyrosine residues that are further dephosphorylated by EYA tyrosine phosphatase activity.

In this paper we show first that two human EYA forms, EYA1 and EYA3 are phosphorylated by Src kinase and both can autodephosphorylate the resulting phosphotyrosine residues. Searching for tyrosine residues which can be potentially phosphorylated by Src kinase, we identified two tyrosine residues which are essential for the catalytic activity of EYA3. Their mutation to phenylalanine generated catalytically inactive forms which are in the same time novel, highly efficient trapping mutants for EYA3. To find novel cytoplasmic substrates of EYA3 we performed a peptide microarray profiling which suggested that cytoskeleton protein WD-repeat-containing protein 1 (WDR1) may serve as potential substrate for its tyrosine phosphatase activity. Further on, we show that WDR1 is phosphorylated by Src kinase. Moreover, we demonstrate that phosphorylated WDR1 interacts and is dephosphorylated by EYA3 in a specific manner, proving that is a *bona fide* substrate of EYA3. Finally, we show that lack of Src-mediated phosphorylation of WDR1 leads to significant changes of cellular actin cytoskeleton. This finding suggests that WDR1 dephosphorylation by EYA3 may play an important regulatory role in the modulation of actin cytoskeleton.

## Results

### Human EYA1 and EYA3 proteins are phosphorylated by Src kinase and both can autodephosphorylate

**Figure 1:**
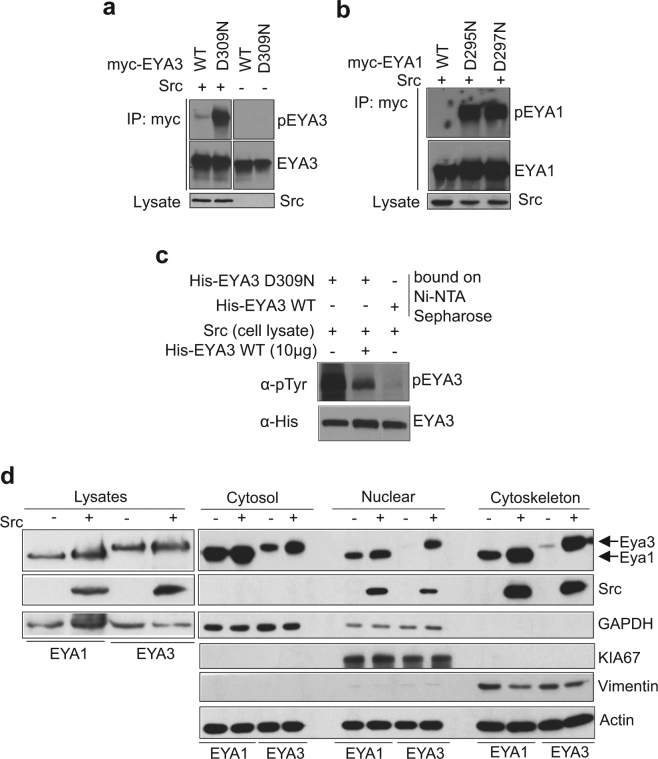
EYA1 and EYA3 proteins are phosphorylated by Src kinase and both can autodephosphorylate. (**a**,**b**) Wild-type EYA1 and EYA3 and their catalytically inactive mutants were co-expressed in 293 T cells with Src kinase. The proteins were immunoprecipitated from cell lysates with anti-myc antibody and analyzed by western blotting with anti-phosphotyrosine and anti-myc antibodies. Whole cell lysates (WCLs) were immunoblotted with anti-Src antibody. Samples from Fig. 1a were processed on the same blot and the full-length blots are presented in Supplementary Fig. S17. (**c**) His-tagged EYA3 WT and D309N mutant bound to Ni-NTA Sepharose beads were incubated with 293 T cell lysate. For lane two, 10 μg of His-tagged EYA3 active protein was added additionally in the lysate. The phosphorylation level of proteins was assessed by western blotting with anti-phosphotyrosine antibody. (**d**) Immunoblot analysis of different subcellular fractions of 293 T cells overexpressing either EYA1 or EYA3 proteins with or without Src kinase. The fractions were analyzed with the subcellular markers: GAPDH (cytoplasm), KIA67 (nuclear), vimentin (cytoskeleton) and actin as loading control for equal protein amounts of all fractions. The images are cropped from original blots (**b**,**c**,**d**).

*Drosophila* EYA phosphatase has the unusual characteristic to interact with itself^17^ and autodephosphorylate^6^. The first questions we aimed to answer were whether human EYA orthologs have the same capacity to autodephosphorylate and if so, which kinase is responsible for the phosphorylation of tyrosine residues of EYA? To address these questions we initially obtained catalytically inactive mutants of EYA1 and EYA3. The catalytically inactive forms of EYA1 and EYA3 were obtained by mutating the essential aspartic residues (D295 and D297 in human EYA1 and D309 and D311 in human EYA3) to asparagine residues^5^. To evaluate the capacity of different tyrosine kinases to phosphorylate EYA protein, we tested Abl, Btk and Src tyrosine kinases. All three kinases phosphorylated mutant EYA3 protein as compared with wild-type EYA3 (EYA3 WT) and untreated samples (Fig. 1a and Supplementary Figs S1, 2). Given the particular significance of Src kinase activation in a variety of cellular processes (in cell proliferation, cytoskeletal reorganization^18^ and in more than 50% of the cases of colon, liver, lung, breast, and pancreas tumors^19^) it was chosen for further, in-depth study. Catalytically inactive mutants of EYA1 can also be phosphorylated by Src kinase as compared with the wild-type protein (Fig. 1b and Supplementary Fig. S2). The reduced phosphorylation signal obtained for wild-type EYA1 and EYA3 proteins as compared to the catalytically inactive forms suggests that both possess autocatalytic activity. To prove this, we used two approaches. First, WT and mutant inactive EYA3 immobilized on Ni-NTA Sepharose were treated with cell lysate containing overexpressed Src kinase. Figure 1c shows that only inactive EYA3 mutant form was phosphorylated (lane 1). Beads containing immobilized EYA3 mutant were also treated with EYA3 WT leading to substantial dephosphorylation. Second, His-tagged EYA3 constructs (Supplementary Fig. S3) were expressed in *E. coli* cells and purified as described in the manuscript under “Materials and Methods”. Next, the proteins were incubated with active Src kinase in the presence or absence of benzbromarone, a specific inhibitor of EYA phosphatase^20^. In absence of benzbromarone the phosphorylation signal for the EYA3 WT and C-terminal domain-containing protein (EYA3ED) was lower than for the inactive full-length EYA3 (EYA3 D309N) and the N-terminal domain (EYA3∆ED) (Supplementary Fig. S4). In contrast, when benzbromarone was used the phosphorylation level increased for both the active forms EYA3 WT and EYA3ED, suggesting that the reduced phosphorylation level is a result of EYA3 autodephosphorylation. Therefore, these experiments showed that Src kinase has phosphorylation sites both in N-terminal and C-terminal domains of EYA3 protein and these sites can be autodephosphorylated.

Our next question was if EYA phosphorylation by Src kinase influences its subcellular localization/distribution, knowing that Abl kinase shuttled phosphorylated EYA between nucleus and cytoplasm^16^. Thus, 293 T cells were transfected with EYA1 or EYA3 with or without Src kinase and the resulting cells were used for subcellular fractionation. Transiently expressed EYA1 and EYA3 proteins were found in all three studied compartments (cytosol, nuclear and cytoskeleton), but the extent of their expression was influenced by Src kinase. Clearly, phosphorylation by Src stimulates nuclear and cytoskeletal localization of both EYA forms. However, while EYA1 is present in a reduced quantity in nucleus and cytoskeleton in absence of Src kinase, the localization of EYA3 in these compartments is controlled in all-or-none manner by Src kinase dependent phosphorylation (Fig. 1d and Supplementary Fig. S5). Thus, the nuclear and cytoskeletal localization of EYA1 and EYA3 proteins seems to be stimulated, respectively strictly controlled by Src phosphorylation.

### Two tyrosine residues from the conserved C-terminal domain control the catalytic activity of EYA3, but not EYA1

**Figure 2:**
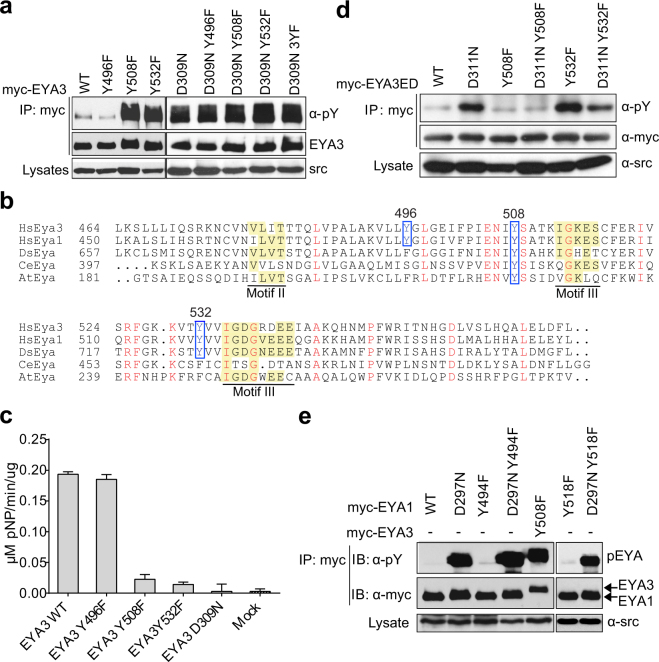
Two tyrosine residues from the conserved domain are essential for the catalytic activity of EYA3. (**a**) myc-tagged EYA3 WT, EYA3 D309N and different tyrosine mutants of both WT and inactive mutant were co-expressed in 293 T cells with Src kinase. The proteins were immunoprecipitated, followed by western blotting with anti-phosphotyrosine and anti-myc antibodies. WCLs were immunoblotted with anti-Src. Samples derive from the same experiment and blots were processed in parallel. (**b**) Alignment of the conserved domain (ED) of human EYA3 (Q99504, 464–573 residues), human EYA1 (Q99502, 450–559), Drosophila EYA (Q05201, 657–766 residues), worm EYA (O17670, 397–502 residues) and plant EYA (O82162, 181–307 residues). Motifs characteristic of HADs family are highlighted with yellow. The strictly conserved residues are marker with red. The residues marked in a blue square were mutated to phenylalanine. Alignment was generated using ClustalW program^25^. (**c**) The histogram is showing the amount of product (pNP) formed in the phosphatase reaction for immunoprecipitated myc-EYA3 proteins when pNPP was used as substrate. Error bars represent the standard deviation from two independent readings. (**d**) 293 T cells were co-transfected with myc-EYA3ED wild-type or mutants (D311N, Y508F, D311N Y508F, Y532F and D311N Y532F) and Src kinase. The immunoprecipitated proteins and WCLs were immunoblotted as indicated. (**e**) myc-EYA1 WT or mutants (D297N, Y494F, Y518F or D297N combination with tyrosine mutations) were co-expressed with Src kinase in 293 T cells. myc-EYA3 Y508F construct was used for comparison. The immunoprecipitated proteins and WCL were analyzed by western blotting as indicated. The images are cropped from original blots (**a**,**d**,**e**).

To identify the phosphorylation targets of Src kinase in EYA3 protein we used several predictors: KinasePhos^21^, iGPS 1.0^22^ and NetPhos2.0^23^. All predictors indicated that there are numerous tyrosine phosphorylation residues located both in the N-terminal and the C-terminal domains. The myc-tagged Y → F mutants, corresponding to the sites with high prediction scores, were transiently co-expressed in 293 T cells with Src kinase, the proteins were immunoprecipitated and their phosphorylation state was determined by immunoblotting. The results indicated that none of the tested tyrosine residues from the N-terminal domain (Y96, Y105, Y208 and Y237) contribute significantly to the EYA3 phosphorylation (Supplementary Fig. S6). Next, we investigated Y496, a potential phosphorylation site revealed by our preliminary mass spectrometry analysis, and the predicted tyrosine residues Y508 and Y532, all from the C-terminal domain. Thus, the analysis of full-length EYA3 proteins containing single point mutations for these tyrosine residues shows that the phosphorylation level of the proteins containing either Y508F or Y532F mutations increased while Y496 mutation slightly decreased compared to EYA3WT (Fig. 2a).These results clearly suggest the functional non-equivalence of tyrosine residues Y508, Y532 and of tyrosine residue Y496. The increased tyrosine phosphorylation level generated by Y508F and Y532F mutations is an unexpected outcome which cannot be simply explained by the tyrosine residues propensity to be phosphorylated by Src kinase. However, reminding that EYA proteins may autodephosphorylate^6^, the results in Fig. 2 could be understood accepting that Y to F mutation of specific tyrosine residues may reduce the autodephosphorylation capacity. This assumption is supported by the comparable level of tyrosine phosphorylation at Y508F or Y532F mutants and the catalytically inactive form D309N **(**Fig. 2a**)**.

Similarly, the double EYA3 mutants (D309N combined with the Y508F or Y532F mutations), presented the same level of phosphorylation like the inactive mutant (Fig. 2a). Moreover, the phosphorylation level was not reduced even in the case of the inactive mutant combined with the triple Y to F mutations (D309N 3YF = D309N Y496F Y508F Y532F) (Fig. 2a). To evaluate the potential functional role of mentioned tyrosine residues on EYA proteins, we performed sequence alignment of EYA domains^24,25^. Alignment shows that Y508 is a highly conserved residue and Y532 is conserved in EYA from mammals and flies (Fig. 2b). All these observations suggest that both Y508 and Y532 residues might influence the phosphatase activity of EYA3. Thus, we performed an *in vitro* PTP assay using immunoprecipitated enzymes and para-nitrophenyl phosphate (pNPP) as an artificial substrate. We observed that Y508F and Y532F mutations lead to catalytically inactive enzymes, both in full-length EYA3 (Fig. 2c) and in EYA3ED proteins (Supplementary Fig. S7). Up to this point we cannot assess whether Y508 and Y532, besides their direct or indirect role in the catalytic activity, are also phosphorylation sites for Src kinase or not. To address this issue, we further checked the phosphorylation level of the ED domain of the EYA3 containing either Y508F or Y532F mutations. Figure 2d shows that the phosphorylation level of Y508F mutant is similar with that of EYA3ED WT. On the other hand, taking into account the phosphatase inactivating role of Y508F mutation (see lanes 1 and 3 in Fig. 2a,c) we can infer that Y508 is a Src-phosphorylation site. EYA3 can autodephosphorylate it, while Y508F mutation abolishes its phosphorylation. Regarding Y532, we may notice that phosphorylation level of Y532F in EYA3ED is similar to the D311N inactive mutant (Fig. 2d lanes 2 and 5) suggesting that Y532 is not a Src-phosphorylation site. Moreover, comparing lanes 1, 3 and 5 in Fig. 2d, it can be assessed that Y508 is the only autodephosphorylation site in the C-terminal EYA3ED.

The investigated tyrosine residues are conserved among mammalian and fly EYA phosphatases (Fig. 2b), thus we further evaluated whether the enzymatic activity of other EYA members is influenced by these two tyrosine residues. In this context, we analyzed the phosphorylation pattern of EYA1 protein containing the corresponding mutations from EYA3 (Y494 corresponds to Y508 and Y518 to Y532). Unexpectedly, the results indicated that, in contrast to EYA3, the mutation of these two tyrosine residues to phenylalanine in EYA1 does not affect its enzymatic activity, the phosphatase still being able to autodephosphorylate (Fig. 2e, Supplementary Fig. S8a,b,c and Supplementary Table S1). Altogether, these findings suggest that Y508 and Y532 directly or indirectly control the catalytic activity of EYA3 protein, while the equivalent tyrosine residues in EYA1 do not have this role. To clarify whether Y496 is or is not a Src-phosphorylation site for EYA3 we performed another experiments to assess the extent of phosphorylation of immunoprecipitated Y496F and some other mutants of EYA3ED. The results presented in Supplementary Fig. S8d show that both Y496F and Y508F exhibit comparable but minor decrease compared to the WT form. However, double mutant EYA3-ED Y496F and Y508F has a considerably lower phosphorylation signal than the WT form. This finding suggests that both Y508 and Y496 are Src-dependent tyrosine-phosphorylation sites of EYA3.

### EYA3Y508F and EYA3Y532F are new trapping mutants for EYA3 phosphatase

**Figure 3:**
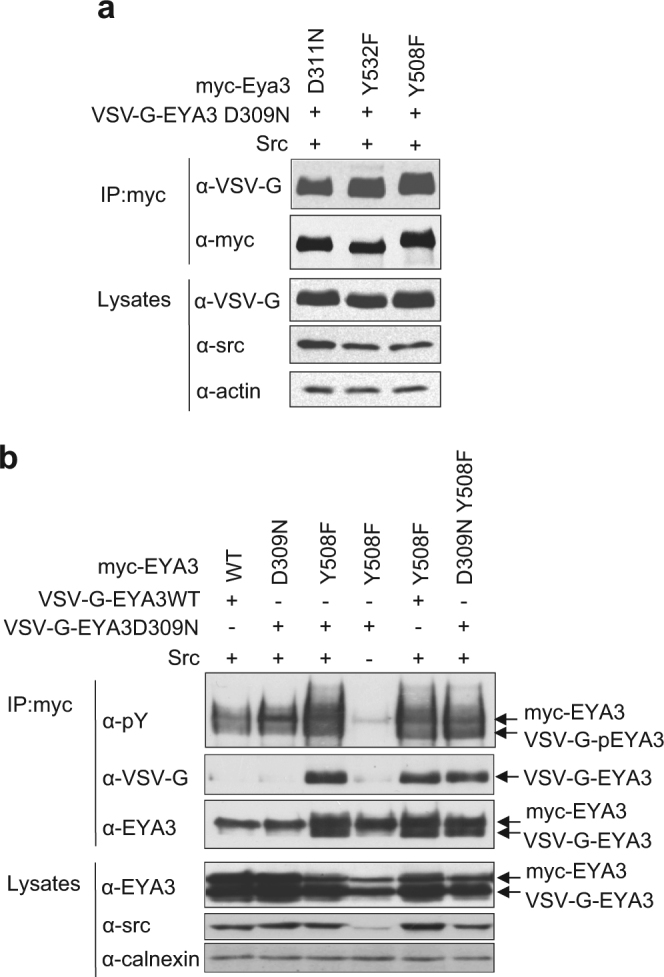
EYA3 Y508F and EYA3 Y532F proteins are new, specific trapping mutants for EYA3 phosphatase. Src kinase was co-expressed in 293 T cells with: (**a**) myc-tagged EYA3 single point mutants (D311N, Y508F and Y532F) and VSV-G-tagged EYA3 D309N. The images are cropped from original blots. (**b**) VSV-G-EYA3 (WT and D309N) and myc-tagged EYA3 (WT, D309N, Y508F and D309N Y508F). EYA proteins were immunoprecipitated from cell lysates and trapped proteins were analyzed by western blotting with indicated antibodies. WCLs were immunoblotted with anti-Src and anti-actin. Full-length blots are presented in Supplementary Fig. S18.

Mutations of both Y508 and Y532 to F abolish the catalytic activity of EYA3 (Fig. 2a,c), therefore we asked whether they abrogate the substrate binding capacity of EYA3 as well. Supposing these mutants still preserve their ability to bind substrates, then the corresponding Y → F mutations may provide novel trapping mutants. Previous evidence^6^ and here reported results show that EYA can dephosphorylate itself. Thus, to test the trapping capacity of the mentioned EYA3 mutants it is sufficient to prove that they can interact with another inactive, tyrosine-phosphorylated EYA mutant. To this end, we co-expressed in 293 T cells pairs of EYA3 proteins labelled with different tags (myc or VSV-G) as well as Src kinase. Then, the myc-tagged proteins were immunoprecipitated and the trapped protein was detected with anti-VSV-G antibody. As shown in Fig. 3a, both myc-EYA3Y508F and myc-EYA3Y532F bind VSV-G-EYA3D309N with similar affinities as myc-EYA3D311N, a known trapping mutant of EYA3. The good trapping capacity of EYA3Y508F is also supported by the fact that the inactive EYA3 construct (EYA3D309N) which has no trapping capacity (Fig. 3b) turned into a good trapping mutant by mutating tyrosine Y508 to phenylalanine (EYA3D309NY508F construct). Further on, we observed that myc-EYA3Y508F binds VSV-G-EYA3D309N inactive mutant only when Src kinase is present, suggesting that the EYA-EYA interaction is phosphorylation-dependent (Fig. 3b and Supplementary Fig. S9).

Our results clearly show that EYA3 interacts with EYA3 and in this context we analyzed whether EYA3 could also interact with its paralog, EYA1. Thus, VSV-G-EYA3 Y508F was co-expressed in 293 T cells with either myc-EYA1 WT or myc-EYA1 D297N (the trapping mutant for EYA1) in presence of Src kinase. Immunoblotting analysis indicated that EYA3 does not interact with EYA1 (Supplementary Fig. S10). This result indicates that EYA3-EYA3 interaction is paralog specific.

### EYA3 phosphatase activity profiling on peptide microarrays

**Figure 4:**
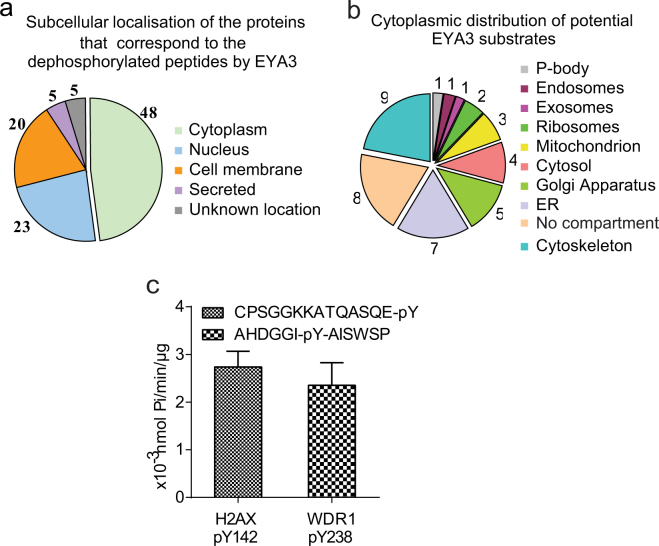
EYA3 phosphatase profiling on peptide microarrays. (**a**) The subcellular localization of the 68 human proteins corresponding to the tyrosine phosphorylated peptides dephosphorylated by EYA3 in the peptide microarray assay. Note: some of the proteins were identified in more than one location inside the cell. (**b**) The distribution of the cytoplasmic proteins that are potential substrates for EYA3 phosphatase. (**c**) Histogram is showing the amount of product formed in the phosphatase reaction for His-EYA3 WT protein when two different peptides were used as substrates. Error bars represent the standard deviation from three independent experiments. CPSGGKKATQASQE-pY peptide corresponds to pY142-H2AX, a known substrate for EYA phosphatase. AHDGGI-pY-AISWSP corresponds to Y238-WDR1, peptide that was identified dephosphorylated in the microarray assay.

To identify new cytoplasmic substrates for EYA, we performed a phosphatase activity profiling on peptide microarrays using bacterially expressed and purified His-tagged EYA3 active protein. The microarray was composed of 6207 pY-containing peptides derived from human tyrosine phosphorylated proteins. Only 68 phosphopeptides were dephosphorylated more than 70% and 7 of them were dephosphorylated with 100% yield. The subcellular distribution of the 68 potential substrates, according to UniProt/Subcellular location (www.uniprot.org) for each protein, shows that 41 of them have cytoplasmic, 20 nuclear and 17 cell membrane localization (note that some of the proteins were identified in more than one subcellular compartment) (Fig. 4a). At the cytoplasmic level, the potential substrates are distributed in different compartments, 9 of them being in the cytoskeleton (Fig. 4b).

EYA phosphatase activity has been shown to be involved in tumor cell migration, invasion and transformation, and these cellular effects are associated with modifications of the actin cytoskeleton^14^. Therefore, we further focused on a phosphorylated peptide derived from the cytoskeletal protein WD repeat-containing protein 1 (WDR1), which was 100% dephosphorylated in the microarray assay. WDR1 is a 66 kDa protein involved in disassembly of actin filaments together with ADF/cofilin family proteins^26–29^. The 13-residues synthetic peptide (AHDGGI-pY-AISWSP) corresponds to 232–244 aminoacid residues of WDR1 protein phosphorylated at tyrosine 238. According to mass-spectrometry analyses this residue was found phosphorylated in many diseases, like leukemia^30,31^, breast^32^, lung^33^ and gastric^34^ cancers. To characterize the preference of EYA3 phosphatase for this phospho-residue, we compared the enzymatic activity of EYA3 on this peptide and on the phosphopeptide corresponding to H2AX pY142 (CPSGGKKATQASQE-pY), a known substrate of EYA phosphatase^10,11^. As shown in Fig. 4c, comparable levels of phosphate reaction product were obtained for both phosphopeptides tested, suggesting that WDR1 protein may be a substrate for EYA3 phosphatase.

### WDR1 is phosphorylated by Src kinase and dephosphorylated by EYA3 phosphatase

**Figure 5:**
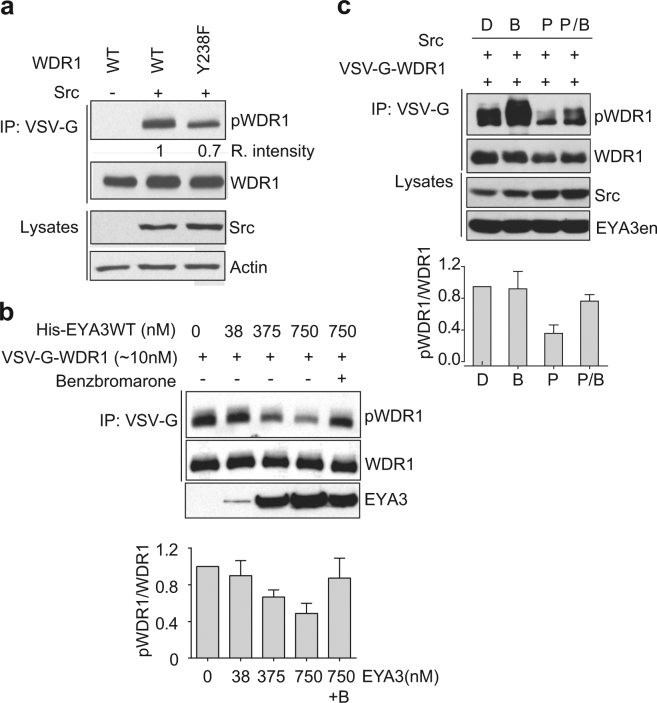
WD40-repeat protein 1 is phosphorylated by Src kinase and dephosphorylated by EYA3 phosphatase. (**a**) VSV-G-WDR1 WT and Y238F mutant were co-transfected with or without Src kinase in 293 T cells. VSV-G-tagged proteins were immunoprecipitated with anti-VSV-G antibody and analyzed by immunoblotting with antibodies for phosphotyrosine (pY) and VSV-G tag. WCLs were analyzed with antibodies against Src and actin. The phosphorylation level of the mutant was evaluated by quantification of signal intensity in western blot using ImageJ software^47^ and compared with WDR1 WT, which was arbitrarily set to 1. (**b**) Equal amounts of immunoprecipitated phospho-WDR1 (pWDR1) protein were incubated with different quantities of recombinant His-EYA3 active phosphatase. In the last reaction, benzbromarone was added in order to inhibit the phosphatase activity of EYA3. WDR1 protein was analyzed by western blotting with antibodies for pY (top) and VSV-G tag (middle). The amount of EYA3 protein used in the experiment was evaluated by immunoblot with anti-His antibody (bottom).The histogram is showing the relative intensity of pWDR1, quantified from 3 independent experiments using Image J by the ratio between pWDR1 and total WDR1 protein. Error bar represent S.D. (**c**) 293 T cells co-expressing WDR1 WT and Src kinase were treated 24 h post-transfection with either DMSO (D), benzbromarone (B), PP2 inhibitor (P) or both PP2 and benzbromarone for other 20 h. Next, WDR1 was immunoprecipitated and analyzed by immunoblotting with antibodies for pY and VSV-G tag. WCLs were analyzed with anti-Src and anti-EYA3 antibodies. The histogram is showing the relative intensity of pWDR1 quantified as in (**b**). Eya3en- eyes absent 3 endogenously expressed in 293 T cells. The images are cropped from original blots (**a**,**b**,**c**).

The phosphatase activity profiling on peptide microarrays indicated that the phosphopeptides derived from WDR1 protein are dephosphorylated by EYA phosphatase, making this protein a potential candidate as EYA3 substrate. Thus, we first obtained tyrosine-phosphorylated WDR1 by co-expressing WDR1 and Src kinase. As shown in Fig. 5a and Supplementary Fig. S11, WDR1 is phosphorylated by Src kinase. In the same time, we analyzed if the Y238 residue from WDR1 was the phosphorylation target of Src kinase. Immunoblotting indicated that the phosphorylation intensity is reduced with 30% in case of WDR1Y238F as compared with WDR1 WT (Fig. 5a). This result suggests that the tyrosine Y238 is phosphorylated by Src kinase, but it is not the unique phosphorylation target.

To prove that WDR1 phosphoprotein is a target for EYA tyrosine phosphatase, we first treated immunoprecipitated WDR1 phosphoprotein with different amounts of EYA3 active protein. As shown in Fig. 5b increasing amounts of EYA3 phosphatase lead to decreased phosphorylation signal of WDR1 phosphoprotein. Addition of benzbromarone^20^, a specific EYA inhibitor in the phosphatase reaction assay, increases the phosphorylation level of WDR1, suggesting that WDR1 dephosphorylation is specifically attributable to EYA3. Second, 293 T cells were co-transfected with WDR1 and Src kinase and 24 hours post-transfection either benzbromarone^20^, Src tyrosine kinase inhibitor PP2^35^ or their combination were added to fresh media. The phosphorylation level of WDR1 was reduced when cells were treated with PP2 suggesting that WDR1 was dephosphorylated by the endogenous EYA3, after Src inhibition by PP2 (Fig. 5c). When PP2 and benzbromarone were simultaneously added, WDR1 phosphorylation was higher than in case when only PP2 was added. These findings strongly suggest that tyrosine phosphorylated WDR1 protein is a substrate for EYA3 phosphatase.

### WDR1 protein specifically interacts only with EYA3

**Figure 6:**
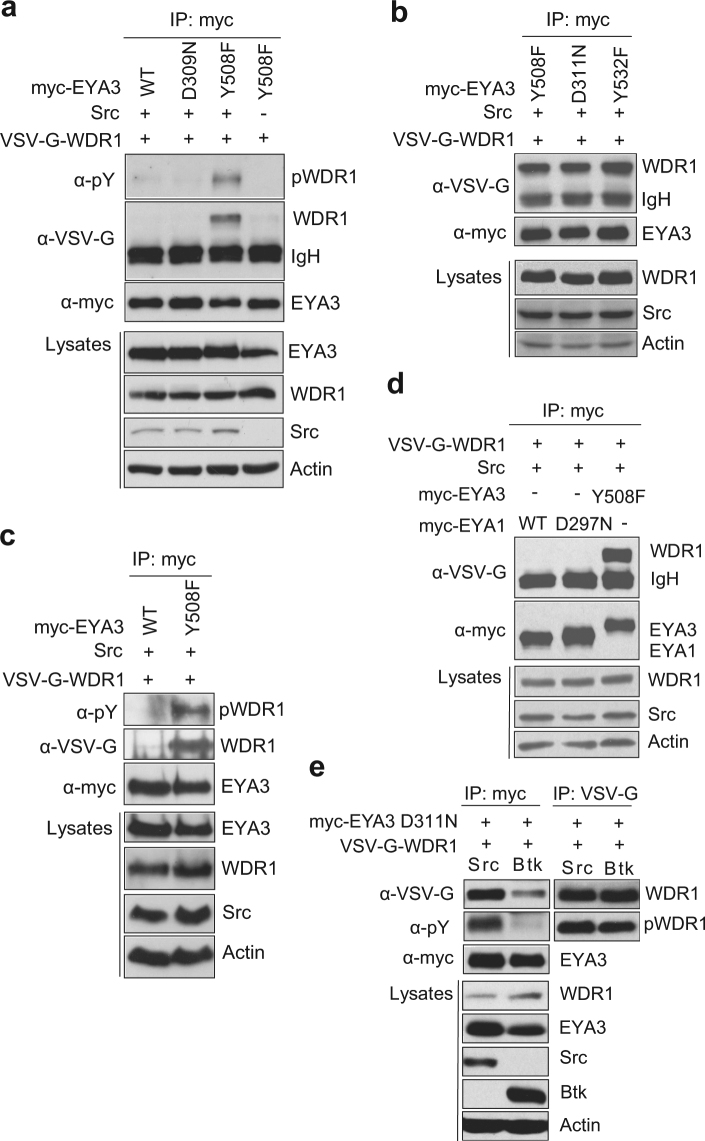
WDR1 cytoskeletal protein interacts only with EYA3. VSV-G-WDR1 WT was co-expressed in 293 T cells with or without Src kinase and different myc-tagged EYA3 constructs (**a**) EYA3 WT, D309N and Y508F; (**b**) EYA3 Y508F, D311N and Y532F. Myc-tagged proteins were immunoprecipitated, followed by western blot analysis with anti-pY, anti-VSV-G and anti-myc antibodies. WCLs were analyzed with antibodies against EYA3, WDR1, Src and actin (bottom). Full-length blots for Fig. 6a are presented in Supplementary Fig. S19. (**c**) MCF7 cells were transfected with VSV-G-WDR1WT and Src kinase with either myc-EYA3 WT or Y508F. Samples were analyzed as described in (**a**) and (**b**). Full-length blots are presented in Supplementary Fig. S20. (**d**) myc-tagged EYA1 WT, EYA1 D297N and EYA3 Y508F were transfected in 293 T cells with VSV-G tagged WDR1 and Src kinase. Samples were further analyzed as described in (**a**) and (**b**).The images are cropped from original blots.(**e**) myc-EYA3 D311N and VSV-G-WDR1 were transfected either with Src or Btk kinase in 293 T cells. Samples were further analyzed as described in (a) and (b). VSV-G tagged proteins were immunoprecipitated followed by western blot analysis with anti-pY and anti-VSV-G. Full-length blots are presented in Supplementary Fig. S21.

To assess whether cytoskeletal protein WDR1 is a substrate for EYA3, we next evaluated the possibility of the interaction between these two proteins. Thus, we co-expressed WDR1 and Src kinase with different myc-tagged constructs of EYA3 and used immunoprecipitation in order to confirm protein interaction. Figure 6a shows that WDR1 protein interacts with EYA3 Y508F trapping mutant, but only in the presence of Src kinase, suggesting that phosphorylation is essential for this substrate-phosphatase interaction. Neither EYA3 WT nor the inactive mutant D309N trap the WDR1 protein, but all trapping mutants of EYA3 (D311N, Y508F and Y532F) immunoprecipitate and have similar affinity for WDR1 (Fig. 6b). Phosphatase activity of EYA has been reported to be involved in actin cytoskeleton changes in breast cancer cell lines^14^. Therefore, we co-expressed EYA3, WDR1 and Src kinase in MCF7 cells (breast cancer cell line) and in Fig. 6c it can be noted that EYA3 interacts with WDR1 in this cell line, too.

Knowing that EYA catalytic domains are highly conserved among EYA family members (EYA 1-4), we further analyzed whether WDR1 protein can also interact with other EYA family members. By immunoprecipitation, we observed that the trapping mutant D297N corresponding to EYA1 does not interact with the cytoskeletal protein WDR1 (Fig. 6d), proving that WDR1 is dephosphorylated in EYA3 -specific manner.

To evaluate the importance of tyrosine phosphorylation for EYA3-WDR1 interaction we tested two kinases: Src and Btk. Figure 6e shows that phosphorylation of WDR1 by Src kinase favors the interaction with EYA3. On the other hand Btk kinase mediated phosphorylation of WDR1 leads to a weak interaction with EYA3 phosphatase (Fig. 6e lane 2).

To further test the capacity of EYA3 to dephosphorylate WDR1, we compared WDR1 tyrosine phosphorylation in MCF7 tumoral cell line treated and untreated with EYA3 specific siRNA. Supplementary Fig. S12 shows that tyrosine phosphorylation of WDR1 is higher in cells with knocked down EYA3. This result adds support to our finding that WDR1 is a specific substrate of EYA3.

### Lack of Src-mediated phosphorylation of WDR1 induces formation of irregularly organized actin fibers

**Figure 7:**
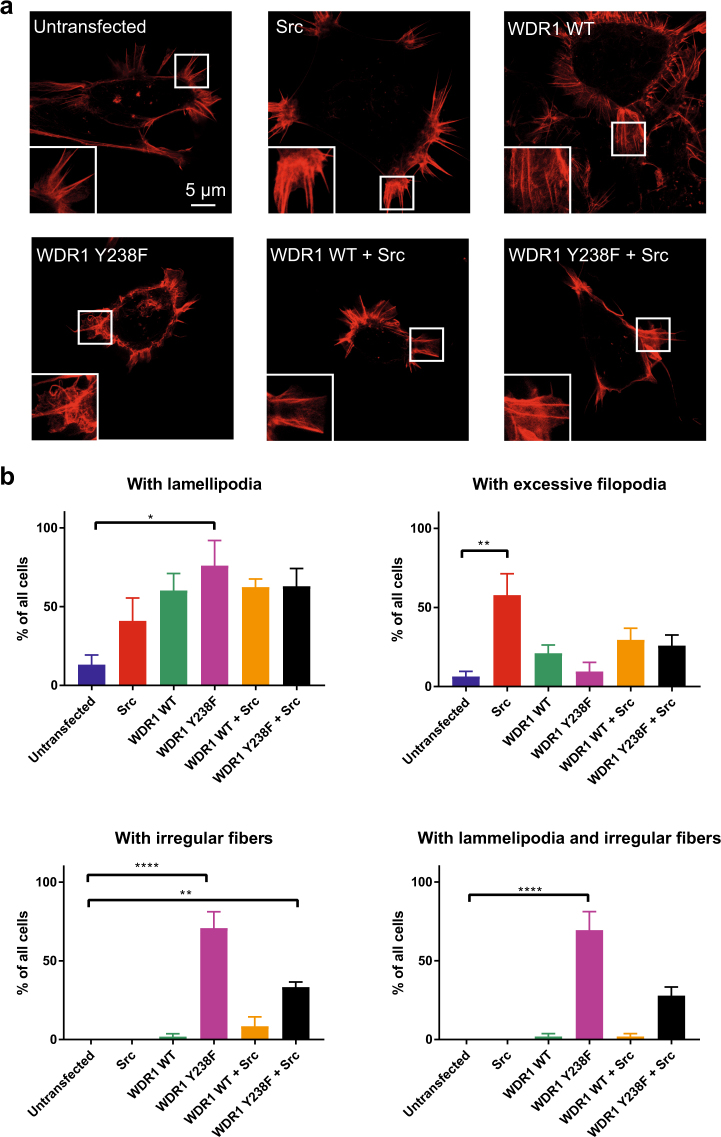
WDR1 expression, and its phosphorylation at the Y238 residue, modifies the actin cytoskeleton. (**a**) Typical images of control (untransfected) cells, or cells transfected with Src kinase, WDR1 wild-type, WDR1 wild-type and Src kinase, WDR1 Y238F, and WDR1 Y238F and Src kinase. The cells were labeled for F-actin, using phalloidin coupled to the dye Star635, and were imaged by STED microscopy. Only cotransfected cells were imaged, assessed by GFP fluorescence (Supplementary Fig. S22). The boxed areas are shown as 2 × zooms, in the insets. Scale bar, 5 µm. (**b**) An analysis of the observed phenotypes. The cells dominated by different phenotypes (excessive amounts of lamellipodia, excessive amounts of filopodia, irregularly organized actin fibers in cell bodies, and irregularly organized actin fibers in the whole cell, including lamellipodia) were counted and the results were expressed as % of all cells. For each category an individual graph is shown. The bars show means ± SEM, from three independent experiments. Significance was assessed by one-way ANOVA, followed by Bonferroni correction. Only significant changes from untreated are indicated in the graphs, for a detailed analysis please refer to Supplementary Tables 3–6.

To examine the role of Src-mediated phosphorylation of WDR1 on the actin cytoskeletal architecture, we investigated by STED microscopy the actin cytoskeleton in 293 T cells transfected with WDR1WT or WDR1 Y238F mutant, each one either co-transfected or not with Src kinase. Significant modifications were observed both in the morphology of specific structural elements of cytoskeleton like filopodia and lamellipodia and in the frequency of cells displaying modifications. Figure 7a shows representative cells for untransformed and transformed cells, while in Fig. 7b the frequency of cells with various modifications is represented. Transfection with the Src kinase increased the number of filopodia, which were rich in F-actin, and were typically assembled in plaques found at 4–6 sites on the cell periphery, plaques which were occasionally indistinguishable from bona fide lamellipodia. Transfection with wild-type WDR1 induced the formation of large numbers of lamellipodia, and a less profound increase in filopodia, which were also less rich in F-actin than those from Src-transfected cells (Fig. 7a). In addition, the cells tended to form filopodia and lamellipodia around the cell circumference, leading to a less polarized morphology (compare with wild-type cells in the first panel of Fig. 7a). This may be due to the lack of phosphorylation of WDR1, since co-expression of WDR1 with Src-kinase corrected this phenotype (Fig. 7a), albeit the overall proportion of cells containing excessive numbers of filopodia or large lamellipodia was indistinguishable from that in cells transfected with WDR1 alone (Fig. 7b). Far more severe modifications of the actin cytoskeleton were observed in cells transfected with WDR1 Y238F. The cells formed large numbers of lamellipodia, but these contained irregularly organized, curled F-actin fibers, which could also be observed in other areas of the cells. This resulted in the lamellipodia having a more irregular shape that in other genotypes. This phenotype was partially repaired by co-expressing WDR1 Y238F and Src. The cells were less rounded, more polarized than those expressing WDR1 alone (wild-type or Y238F), and contained ~50% fewer irregularly organized F-actin fibers. This supports the conclusion that the absence of Src-dependent phosphorylation of Y238 generates irregularly organized fibers. At the same time, increasing the phosphorylation of WDR1 at other tyrosine residues, by expressing Src, has the potential of partially rescuing this phenotype. Knockdown of WDR1 and also EYA3 affect the actin cytoskeleton in MCF7 cells as well (Supplementary Figs S13 and S14).

Overall, these experiments suggest that the expression of WDR1 (wild-type or Y238F) is sufficient to cause excessive levels of lamellipodia, and also filopodia (to a smaller level). In addition, the inability to phosphorylate WDR1 at the Y238 residue leads to irregularly organized actin fibers.

To get insight into the role of phosphorylated WDR1 in a tumor cell line, the proliferation rate of MCF7 overexpressing WDR1 WT and WDR1 Y238F with or without Src kinase was analyzed (Supplementary Fig. S15). Comparing the proliferation rates of MCF7 cells not transfected with Src suggests that WDR1 overexpression negatively contributes to the proliferation while single point mutation Y238F of WDR1 substantially increase it. On the other hand phosphorylation of WDR1 seems to have a positive contribution to the proliferation (see cells transfected with WDR1 WT with or without Src). Taking into account the similar proliferation rates of cells transfected with WDR1 Y238F with or without Src we can infer that phosphorylation of tyrosine residues others than Y238 plays a minimal role in the overall stimulation of proliferation by tyrosine phosphorylation.

## Discussion

Here, we report that Src non-receptor tyrosine kinase can phosphorylate both human EYA1 and EYA3 proteins. The activation of the c-Src pathway has been observed in about 50% of the tumors from colon, liver, lung, breast and the pancreas^19^. It has been suggested that the oncogenic potential of Src resides in the activation of a number of key signaling molecules involved in cellular pathways^36^. Accounting that members of EYA family are also overexpressed in various forms of cancer^1^, thus promoting proliferation, transformation, migration and invasion of tumor cells^14^, we suggest that overexpression of Src and EYA in given cancer forms may be linked during oncogenic development.

The experiments involving EYA3 mutants showed that Src kinase phosphorylates multiple tyrosine phosphorylation sites in the N-terminal domain and two in the C-terminal catalytic domain (Y496 and Y508). Identification of all tyrosine residues phosphorylated by Src kinase and potential role of tyrosine-phosphorylation needs further investigations. Abrogation of EYA3 phosphatase activity by Y508F and Y532F mutations is an unexpected outcome of these experiments. A reasonable explanation is that the mentioned mutations induce conformational modifications in the structure of EYA3 molecule which ultimately lead to the cancellation of its catalytic activity and implicitly of its autodephosphorylation capacity (Supplementary Fig. 16)^37^. The mentioned conformational modifications do not alter EYA3 substrate-binding ability and thus, Y508F and Y532F are efficient substrate-trapping mutants. A similar effect has been reported for a classical type PTP, Shp2, where T466A mutation has been proven to abolish phosphatase activity^38^. The novel EYA3 trapping mutants Y508F and Y532F reported here provide important molecular tools for identification of new physiological substrates. Notably, because the equivalent tyrosine residues in EYA1 do not have similar effect on the catalytic activity, it can be expected that the new substrate trapping mutants can be used for identification of EYA3-specific substrates.

Various reports support the subcellular localization of EYA proteins both in cytoplasm and the nucleus^1,16^. It has been also reported that Abl kinase-mediated phosphorylation recruits EYA to cytoplasm^16^, while cytoplasmic EYA translocates to the nucleus upon interaction and formation of a transcriptional complex with SIX1 homeodomain protein^13^. Data presented here show that Src-mediated phosphorylation of EYA1 and particularly of EYA3 controls the subcellular localization to cytoskeleton and nucleus. On the other hand, the interplay between the Src-mediated phosphorylation and autodephosphorylation provides an optimal balance between the phosphorylated and unphosphorylated forms of EYA.

As EYA paralogs are able to autodephosphorylate, it is reasonable to ask whether one EYA could dephosphorylate another EYA. The lack of immunoprecipitation of EYA3 by EYA1 suggests that the two proteins do not interact (Supplementary Fig. S10). Therefore, the reciprocal dephosphorylation between paralogs, at least between EYA1 and EYA3 is not possible. This finding shows that autodephosphorylation is also paralog specific and cross-regulation of catalytic activity/subcellular localization between EYA paralogs cannot take place.

One of the very few validated substrates for the tyrosine phosphatase activity of EYA is DNA damage-related histone H2AX^10,11^. Both EYA1 and EYA3 are able to dephosphorylate H2AX^10^. However, the results reported here, showing that WDR1 is a substrate of EYA3, but not of EYA1 are unanticipated and suggest that minor sequence difference between EYA forms may induce major modifications of substrate specificities against the same phosphotyrosine-containing protein. This fact complies with the observations that mice deficient in one of the four EYA paralogs display essentially different phenotypes and that each protein play distinct physiological roles^1^.

Examination of the STED microscopy images regarding the impact of different WDR1 constructs on the cytoskeletal architecture reveals particularities that were unusual in normal cells, including the presence of a multitude of lamellipodia/filopodia (for WDR1 WT and partly for WDR1 WT + Src), or a chaotic overall aspect, both of which are reminiscent of tumoral/metastatic cells^39^ (Fig. 7a). Remarkably, the stabilized segments of cortical F-actin were observed in untransfected, Src-transfected and partly in WDR1 Y238F + Src – transfected cells. The presence of stabilized cortical F-actin has been reported to be a feature of cells with reduced motility and invasiveness^39^. Regarding the abundance of lamellipodia and of filopodia in cells co-transfected with WDR1 WT and Src (Fig. 7a), it resembles the organization of migrating cells. This finding is supported by previous reports showing that WDR1 is upregulated in highly metastatic cells in gallbladder carcinoma^40^, in invasive ductal carcinoma^41^ or in primary glioblastoma^42^. As concerning the irregularly organized fibers, observed first of all in cells transfected with the Y238F mutant form of WDR1, we cannot assess exactly to what type of actin protrusions they belong to. However, they are closest in morphology and intensity of fluorescent labeling to filopodia, which may have become modified morphologically due to perturbed actin dynamics. This is possibly correlated with changes at the level of other proteins involved in formation of filopodia like cofilin, fascin or formins. Cofilin, which has been detected in the filopodia of several cell types and proved to contribute to disassembling of filopodial actin filaments^43,44^ may play an important role in this respect.

WDR1 is, up to our knowledge, the first reported cytoplasmic substrate of EYA3 tyrosine phosphatase and this finding is in agreement with EYA’s documented role in cell motility^14^. WDR1’s physiological importance has been evidenced since long by siRNA-mediated silencing in HeLa and Jurkat T-lymphoma cells^26^. These knockdown experiments proved that WDR1 plays critical roles in directional cell migration by restricting the membrane protrusion via promoting cofilin activity. According to a recent report, it facilitates disassembly of cofilin-saturated actin filaments by severing and accelerating monomer dissociation from barbed and pointed ends^45^. Interestingly, the authors of this paper found that WDR1 does not displace cofilin from F-actin, but rather induces actin filament depolymerization at cofilin occupancies. A simple model can be advanced to explain our results regarding WDR1 implication on cytoskeleton. Overexpression of WDR1 WT generates numerous filopodia (Fig. 7). However, Y238 residue of WDR1 is mostly unphosphorylated and just a small number of them are phosphorylated by the constitutive, cellular Src kinase. Assuming that phosphorylation of Y238 stimulates the ternary interaction between WDR1-cofilin-actin then it is reasonable to accept that WDR1 with low extent of phosphorylation at Y238 does not stimulate the cofilin-dependent disassembly of actin filament. Consequently, cofilin-severing of actin filaments in cells transfected with WDR1 WT is reduced, so that filopodial retraction is hindered, existing filopodia remain in stasis and formation of novel protruding filopodia is limited leading to the multitude of filopodia uniformly distributed on the surface of non-polarized cells. However, when the cells are transfected with the Y238F mutant form of WDR1 instead of WDR1 WT, the phosphorylation of Y238 is completely suppressed. As a consequence, the WDR1-cofilin interaction is severely compromised leading to a much smaller number of filopodia, formation of irregularly organized fibers and disordered cytoskeletal architecture. Co-expression of Src with WDR1 Y238F rescues the interaction with cofilin, probably due to the Src-dependent phosphorylation of tyrosine residues other than Y238.

In conclusion, we identified WDR1 as a specific, cytoplasmic substrate of EYA3. Phosphorylation of Y238 and of other tyrosine residues of WDR1 is an important molecular tool for modulation of the actin cytoskeleton. EYA3 emerges as a key player in maintaining an optimal balance of the phosphorylation level of these tyrosine residues. Thus, our results provide a novel basis for understanding the role of EYA tyrosine phosphatase activity in modulating the cytoskeletal architecture of the cell.

## Materials and Methods

### Antibodies

Anti-myc tag (clone4A6) for immunoprecipitation, anti-v-Src (clone 327) were purchased from Merck Millipore and Thermo Fisher; c-Myc(9E10) HRP for western blotting, VSV-G(P5D4), WDR1(G-13), anti-calnexin, p-Tyr(PY99) HRP, anti-vimentin, anti-GAPDH and secondary antibodies coupled with HRP from Santa Cruz Biotechnology Inc; Anti-WDR1 Atlas Antibodies; Anti-Actin Ab-5 (Clone C4/actin) from BD Biosciences; Anti-EYA3 from Abcam, Proteintech and Atlas Antibodies, anti-calnexin from Abcam; GST-tagged active v-Src kinase protein from SignalChem; Anti-His antibody from GE Healthcare.

### Constructs

The cDNA sequences of both human EYA1 (Q99502) and human EYA3 (Q99504) were amplified by PCR from the Human Transcriptome cDNA library from Stratagene. EYA1 sequence was inserted into pCSMT2 vector and EYA3 was inserted into pCS-MT2 (myc-tag), pCineo (VSVG-tag) and pHAT2 (6xHis-tagged) vectors. cDNA of human WDR1 was amplified form pEYFP-Aip1/WDR1 construct (kind gift from Prof. K. Mizuno, Tohoku University, Sendai, Japan.) and subcloned into pCineo vector (kind gift from Dr. V. Ivan, Institute of Biochemistry, Romania). All EYA and WDR1 mutants were generated by site-directed mutagenesis using QuickChange kit (Stratagene) according to the manufactured instructions. pSlx-SrcY527F, which is expressing a constitutively active Src kinase, and pCSMT2 vectors were a kind gift from dr. Vacaru A.M. (University Medical Center Utrecht, The Netherlands). pcDNA3-Abl-His6-FLAG was a gift from Benjamin Turk (Addgene plasmid # 52684). pCMV-Btk vector was bought from OriGene (myc-DDK-tagged Btk).

### Cell Culture and Transfection

Cells (HEK293T and MCF7) were cultured in DMEM, GlutaMax^TM^ supplemented with 10% heat-inactivated FBS, 1 mM sodium pyruvate and 1 × NEEA. Briefly, cells were seeded in either 6-well plates or 25/75 cm^2^ flasks. One day later, cells were transfected with the indicated plasmids using polyethylenimine (PEI) as transfection reagent (the ratio PEI: DNA was 3:1). In case of phosphorylation experiments, the ratio between plasmid DNA coding for EYA or WDR1 and SrcY527F was 5:1. The ratio between EYA, WDR1 and Src-coding plasmids was 2:2:1.

### Immunoprecipitation

The experiments were performed 24–48 h post-transfection. Cells were washed two times with cold PBS and lysed in 50 mM Tris-HCl, pH 7.4, 150 mM NaCl, 1% NP-40, 10 mM MgCl_2,_ 1 mM ortovanadate, 5 mM iodacetic acid, 1 mM PMSF, 10% glycerol using a syringe with a narrow-gauge needle. The cellular extract was collected by centrifugation at 14,000 × *g* for 25 min at 4 °C and incubated with either 1.5 µg antibody/ 300 µg total protein (for phosphorylation experiments) or 4 µg antibody/1 mg total protein for 1 h at 4 °C, followed by addition of 10 µl of rec-Protein A G-Sepharose and incubation for 16 hr at 4 °C on a rotator. The beads were recovered by centrifugation and washed three times with 50 mM Tris-HCl, pH 7.4, 150 mM NaCl, 10 mM MgCl_2,_ 10% glycerol_._ Finally, the immunoprecipitated proteins were eluted with SDS sample buffer by incubating for 5 min at 100 °C. The eluted proteins were resolved in SDS-PAGE gels and further analyzed by western blotting using the appropriate detection antibodies as indicated in figures.

### Purification of EYA3 proteins

The His-tagged EYA3 fusion proteins were overexpressed in *E. coli* (BL21(DE3)RIL strain) for 15 h at 30 °C, following induction with 0.3 mM IPTG. The collected cells were lysed by sonication in 50 mM Tris-HCl pH 7.4, 350 mM NaCl, 20 mM imidazole, 2 mM MgCl_2_, 0.1% Triton-X-10, 2 mM DTT and 1 mM PMSF. The recombinant EYA3 proteins were purified using an ÄKTA Prime Liquid chromatography system by three steps. First step, metal ion affinity chromatography (HisTrap HP column) using 50 mM Tris-HCl pH 7.4, 350 mM NaCl, 500 mM imidazole, 2 mM DTT, 2 mM MgCl_2_, and 0.1% Triton-X-100 as elution buffer. Second, anion-exchange chromatography (MonoQ 5/50GL column) using 50 mM Tris-HCl pH 7.4, 1 M NaCl, 2 mM DTT, and 2 mM MgCl2 as elution buffer. Last step was size exclusion chromatography (Superdex 75 10/300 GL) using 50 mM Tris-HCl pH 7.4, 100 mM NaCl, 2 mM DTT, 2 mM MgCl_2_ buffer. The proteins were stored at –80 °C in size exclusion chromatography buffer supplemented with 10% glycerol.

### *In vitro* EYA3 phosphorylation and autodephosphorylation assay

4 µg of His-tagged wild-type and mutant EYA3 proteins were bound to 20 µl Ni-NTA Sepharose resin for one hour at 4 °C and then washed 3 times with 20 mM HEPES, pH 7.0, 10 mM MgCl_2_ and 2 mM DTT. Next, Ni-NTA bound proteins were incubated at 4 °C with 1 mg of 293 T cell lysate overnight on a swing rotator. In one sample, containing the mutant EYA3, an additional amount 10 µg of His-tagged EYA3 active protein was added. Further on, the phosphorylation of EYA3 proteins was assessed by western blotting using anti-phosphotyrosine antibody. Two independent experiments were performed.

### Subcellular fractionation

Subcellular Protein Fractionation Kit for Cultured Cells (Thermo Fisher Scientific) was used to obtain proteins from different compartments of 293 T cells transiently co-expressing either EYA1 or EYA3 with or without Src kinase. 48 h post-transfection the cells were collected from 25 cm^2^ flasks by scraping in 1.5 ml cell culture phosphate-buffered saline. 200 µl of cell suspension was saved to control the transfection rate (input) and the remaining cell suspension was further used according to manufacturer’s instructions. Each subcellular protein fraction was quantified and 4ug of each was separated by SDS-PAGE and analyzed by western blot using the appropriate detection antibodies as indicated in figures. The experiment was repeated three times.

### *In vitro* PTP assay

The reactions were performed according to the protocol described by Lorentz U^46^. Briefly, EYA3 wild-type and mutant proteins obtained by immunoprecipitation were washed two times with 20 mM HEPES pH 7 buffer containing 10 mM MgCl_2_ and two times with phosphatase assay buffer (20 mM MES pH 6.5, 100 mM NaCl, 10 mM MgCl_2,_ and 5 mM DTT). Each sample was divided in two equal aliquots and incubated with 10 mM pNPP in 100 µl phosphatase assay buffer, final volume. After 30 min at 37 °C with mixing, the reactions were stopped by adding 300 µl of 3 N NaOH. After 5 min, tubes were spun down for 2 min at 13000 rpm and the absorbance of the supernatant was read at 405 nm in a 96-well microplate using the microplate reader FLUOstar Omega (BMG Labtech). The amount of product, p-nitrophenol, was determined by reading the absorbance at 405 nm and using a molar extinction coefficient of 18,000 M^−1^ cm^−1^. The experiment was performed twice.

### EYA3 phosphatase profiling on peptide microarrays

Purified full-length 6xHis-EYA3 active protein (storage buffer 50 mM Tris, pH7.4, 50 mM NaCl, 2 mM MgCl_2_, 2 mM DTT, 15% glycerol) was used for evaluation of substrate specificity. Peptide microarrays were produced by JPT Peptide Technologies (Berlin, Germany), and the experiments were performed by the same company. Enzyme was used at final concentration of 10 µg/ml in 200 µL 25 mM imidazole buffer pH 6.5, 10 mM MgCl_2_ and 2 mM DTT. The experiment was done once due to the high cost of the microarrays.

### Peptide phosphatase assays

Phosphatase activity against phosphopeptides was measured using Biomol Green (Enzo Life Science). The reactions were performed in 20 mM MES pH 6.5, 100 mM NaCl, 10 mM MgCl_2,_ 5 mM DTT (phosphatase assay buffer) and 50 µM peptide and were initiated by addition of 0.2 µM His-hEYA3.After 40 min at 37 °C 100 µL BG was added and reactions were further incubated 30 min at RT. Phosphate release was measured at 620 nm using the microplate reader FLUOstar Omega (BMG Labtech). The experiment was performed in triplicate and repeated three times.

### *In vitro* dephosphorylation of WDR1 by EYA3

VSV-G-WDR1 WT and Src kinase were co-expressed in 293 T cells for 48 h and then, the phosphorylated WDR1 protein was immunoprecipitated according with the method described above. The immunoprecipitated protein (aprox. 20 nM) was washed two times with lysis buffer and two times with phosphatase assay buffer. Further on, the protein was divided into five equal aliquots and each aliquot was treated with different concentration of His-EYA3 (0, 38, 375 and 750 nM). The final volume of each reaction was 400 µL adjusted with phosphatase assay buffer. The reactions were done at room temperature for 1 hour on a swing rotator. As control, one of the aliquots was incubated both with EYA enzyme (750 nM) and 1 mM benzbromarone. Three independent experiments were performed and the bands corresponding to WDR1 were quantified using ImageJ software^47^. The ratio between relative phosphorylation levels of WDR1 normalized to the total immunoprecipitated protein was represented in a histogram. Error bars represent S.D.

### *In vivo* dephosphorylation of WDR1 by EYA3

VSV-G-WDR1 WT and Src kinase were co-expressed in 293 T cells. Twenty four hours post-transfection the condition media was changed, and cells were mock-treated (DMSO) or treated with either 10 µM Benzbromarone, 10 µM PP2 or together as indicated. After twenty hours, cells were lysed in 50 mM Tris-HCl, pH 7.4, 150 mM NaCl, 1% NP-40, 10 mM MgCl_2,_ 1 mM orthovanadate, 5 mM iodacetic acid, 1 mM PMSF, 10% glycerol and WDR1 protein was immunoprecipitated as described before. Sepharose-bound protein was washed three times with lysis buffer without 1%NP-40. Proteins were eluted with SDS sample buffer and boiled 5 min at 100 °C, and further analyzed by western blotting using the appropriate detection antibodies as indicated in figures. Three independent experiments were performed. The western blot bands were quantified using ImageJ software^47^ and the ratio between the relative phosphorylation level of WDR1 normalized to the total immunoprecipitated protein was represented in a histogram. Error bars represent S.D.

### STED microscopy

The experiments were performed using a Leica TCS SP5 STED microscope (Leica Microsystems, Mannheim, Germany), through a 100 × , 1.4 NA PL APO CS oil objective (Leica Microsystems), as described^48^; or using a modified Quad Scan STED Microscope (Abberior) with a 100 × , 1.4 NA UPlanSApo oil objective (Olympus). Actin was labeled using ATTO647N phalloidin (Sigma Aldrich), using a previously published protocol^49^. ATTO647N was excited using a pulsed diode laser at 635 nm (Leica Microsystems), and depleted using a 750 nm wavelength, provided by a Spectra-Physics Mai Tai tunable laser (pulsed at 80 MHz; Titanium Sapphire, Newport Spectra-Physics, Irvine, CA). For all stainings 3 independent experiments were analyzed and the total number of cells are presented in Supplementary Table S2.

### siRNA mediated knockdown

The experiments were performed using Lipofectamine RNAiMAX (Thermo Fisher), according to the manufacturer’s instructions. Briefly, a combination of 3 siRNAs, each at 10 pmol, targeted against either WDR1, EYA3 or mock were prepared in 12 well plates. MCF7 cells were then seeded at a concentration of 100.000 cells/mL per well. Cells were processed after 48 h by either PFA fixation for immunostaining, or harvested in PBS, supplemented with PMSF and HALT protease inhibitor cocktail (Thermo Fisher) for WB analysis.

### Immunostaining

Cells were fixed with 4% PFA, quenched and permeabilized with PBS with 2.5% BSA and 0.1% Triton. They were then incubated for 1 h each for primary and secondary antibodies. Finally, they were embedded in Mowiol.

### Proliferation Assay

The proliferation assay was carried out using CellTiter 96 AQueous One Solution Cell Proliferation Assay (Promega). MCF7 cells were transfected with pCIneo alone, WDR1 WT or WDR1 Y238F with or without Src kinase using PEI in 6 well plates. 24 hours post-transfection, cells were counted and plated at 10000 cells in 100 µl of DMEM –GlutaMax supplemented with 10% FBS, 1 mM sodium pyruvate and 1 × NEAA per well in 96-well plate. The left over cells were further analyzed by western blot. The cells from 96-well plate were further grown for an additional 48 hours, and the assay was carried out using the manufacturer’s specifications. The A_490_ values were determined using FLUOstar Omega (BMG Labtech). The experiment was performed in duplicate with 3 replicates per experiment.

## Electronic supplementary material

Supplementary information
