# Author Correction: WDR1 is a novel EYA3 substrate and its dephosphorylation induces modifications of the cellular actin cytoskeleton

**DOI:** 10.1038/s41598-020-65507-x

**Published:** 2020-05-20

**Authors:** Mihaela Mentel, Aura E. Ionescu, Ioana Puscalau-Girtu, Martin S. Helm, Rodica A. Badea, Silvio O. Rizzoli, Stefan E. Szedlacsek

**Affiliations:** 1 Department of Enzymology, Institute of Biochemistry of the Romanian Academy, Spl. Independentei 296, Bucharest, 060031 Romania; 2 Department for Neuro- and Sensory Physiology, University Medical Center Göttingen, and Center for Nanoscale Microscopy and Molecular Physiology of the Brain, Cluster of Excellence 171, Humboldtalle 23, Göttingen, 37073 Germany; 3 Max-Planck Research School Molecular Biology, Göttingen, 37077 Germany

Correction to: *Scientific Reports* 10.1038/s41598-018-21155-w, published online 13 February 2018

The Article contains errors.

The image for Lysates/WDR1 in Figure 6B is a duplication of another image from Figure 3A. Additionally, images for Lysates/Src and Lysates/Actin in Figure 6B show incorrect sections of the membrane. The correct Figure 6B appears below as Figure 1.

**Figure 1:**
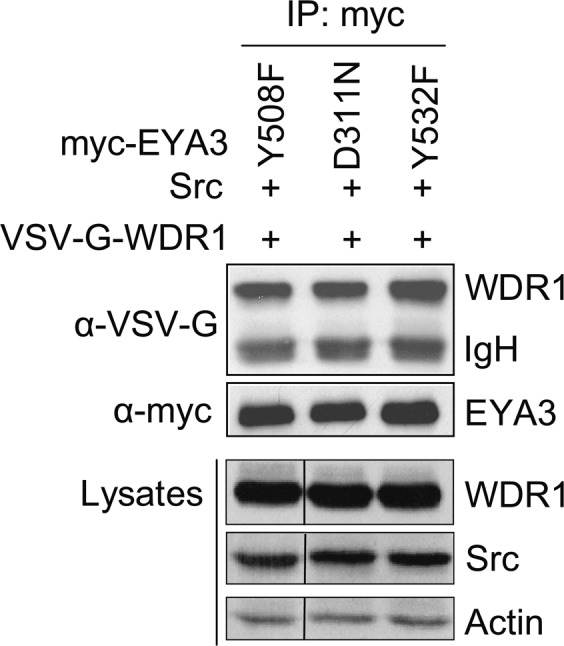
.

The original gels for Lysates samples appear below as Figure 2.

**Figure 2:**
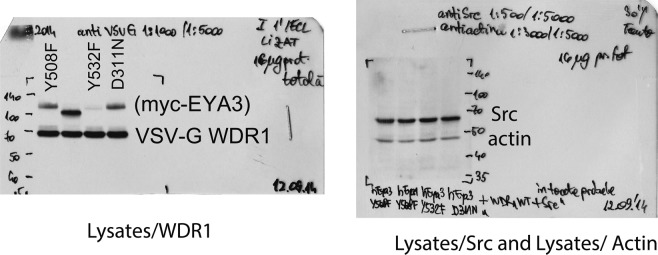
.

The correction does not affect the conclusions of the Article.

