# Supplementary material for: WDR1 is a novel EYA3 substrate and its dephosphorylation induces modifications of the cellular actin cytoskeleton
Source: Sci Rep. 2018 Feb 13;8:2910. doi: 10.1038/s41598-018-21155-w (PMC5811557; doi:10.1038/s41598-018-21155-w)
Supplement: Supplementary file 1 — Supplementary information [file 41598_2018_21155_MOESM1_ESM.pdf]

Supplementary Information File

WDR1 is a novel EYA3 substrate and its dephosphorylation induces modifications of the cellular actin cytoskeleton

Mentel M.[1], Ionescu A. E.[1], Puscalau-Girtu I.[1], Helm M.[2,3], Badea R.A.[1], Rizzoli S.O.[2], Szedlacsek S.E.[1]*

[1]Department of Enzymology, Institute of Biochemistry of the Romanian Academy, Spl. Independentei 296, Bucharest 060031, Romania

[2]Department of Institute for Neuro- and Sensory Physiology, University Medical Center Göttingen, and Center for Nanoscale Microscopy and Molecular Physiology of the Brain, Cluster of Excellence 171, Humboldtalle 23, Göttingen, 37073, Germany

[3]Max-Planck Research School Molecular Biology, Göttingen, 37077, Germany

***For correspondence:** stefan.szedlacsek@biochim.ro

Supplementary methods

Myc-tagged EYA1 and EYA3 phosphorylation assay *in vitro*. EYA1 and EYA3 were immunoprecipitated from 293T cells overexpressing myc-tagged EYA proteins and further incubated with or without 0.128 µg active His-Src kinase (Millipore) in 50 mM MES, 2 mM MgCl2, 2 mM DTT and 100 µM ATP for 1h at 30°C. The phosphorylation level of EYA proteins was analysed by western blot using anti-phosphotyrosine antibody.

6xHis-EYA3 phosphorylation and autodephosphorylation assay *in vitro*. Recombinant His-tagged EYA proteins were *in vitro* phosphorylated by active GST-Src kinase (SignalChem) according with manufactured instructions. Briefly, 2 µg of EYA proteins were incubated with 0.1ug active Src kinase in kinase buffer assay (25 mM MOPS, 12.5 mM β-glycerol-phosphate, 20 mM $MgCl_2$, 25 mM $MnCl_2$, 5 mM EGTA, 2 mM EDTA supplemented with 0.25 mM DTT and 60 nM ATP). The reaction mixture (50 µl) was incubated at 30ºC for 30 minutes. In order to prove the autodephosphorylation capacity of EYA, samples were treated with or without 100µM benzbromarone.

Supplementary Figures

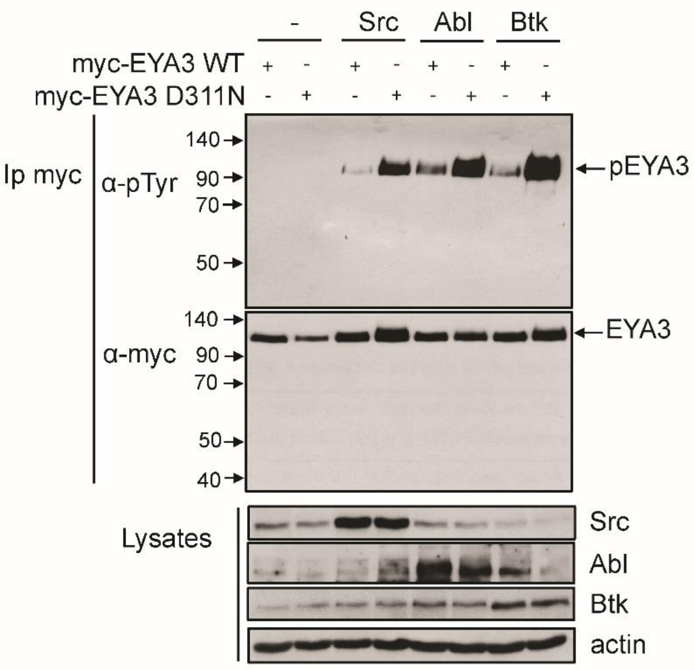

Supplementary Figure S1. Src, Abl and Btk tyrosine kinases phosphorylates EYA3 inactive mutant. Both EYA3 WT and inactive mutant D311N were imunoprecipitated from 293T cells overexpressing Src, Abl or Btk kinase using myc antibody. The phosphorylation state was analyzed by western blot with anti-phosphotyrosine antibody. Whole cell lysates (WCLs) were analyzed with indicated antibodies.

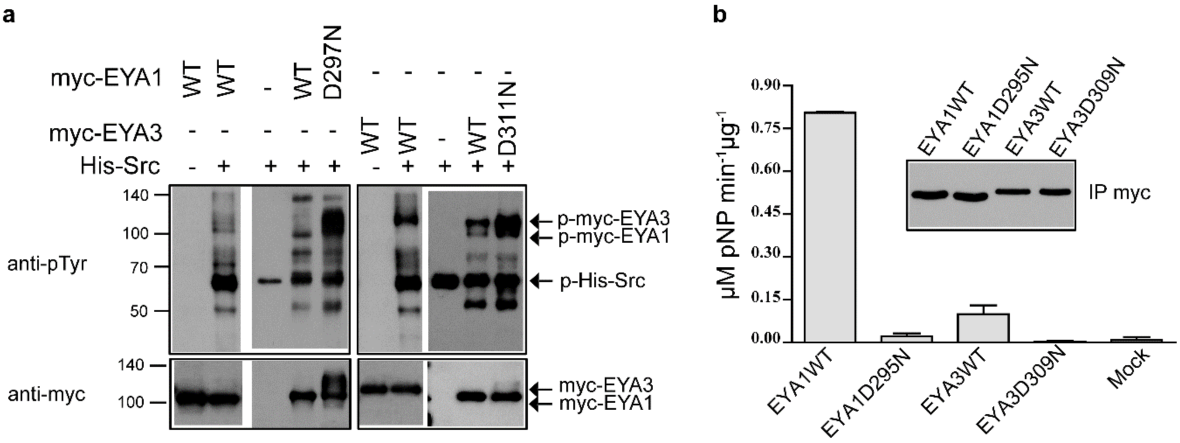

Supplementary Figure S2. EYA1 and EYA3 are phosphorylated by Src kinase *in vitro*. a) Myc-tagged wild-type and mutant EYA1 and EYA3 proteins were immunoprecipitated from 293T cells using anti-myc antibody and further incubated w/o active His-Src kinase (Millipore) for 1h at 30°C in kinase buffer assay. The phosphorylation state was analyzed by western blot with anti-phosphotyrosine antibody. **b)** The histogram is showing the amount of product (pNP) formed in the phosphatase reaction for immunoprecipitated myc-EYA1 and myc-EYA3 proteins when pNPP was used as substrate. Error bars represent the standard deviation from two independent readings. In order to check the amount of the EYA1 and EYA3 proteins used for the enzymatic activity, the immunoprecipitated proteins were further analysed by western blotting. Supposedly, the weaker phosphorylation signal for EYA1 WT compared to EYA3 WT may be explained by a higher catalytic activity of EYA1 than of EYA3 (and implicitly, a higher autodephosphorylation capacity). Testing the catalytic activities of immunoprecipitated, catalytically active and inactive forms of EYA 1 and EYA3 on pNPP proves this hypothesis.

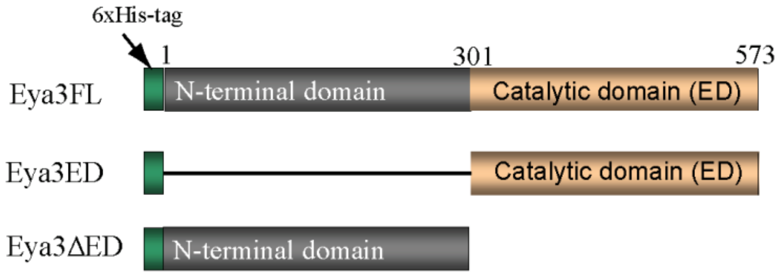

Supplementary Figure S3. EYA3 constructs. Human EYA3 full-length (Q99504, 1-573 residues), the C-terminal domain (EYA3ED, 301-573 residues) and N-terminal domain (EYA3ΔED, 1-300 residues), all three constructs were obtained with Histidine (His)- tag at the N-terminal end.

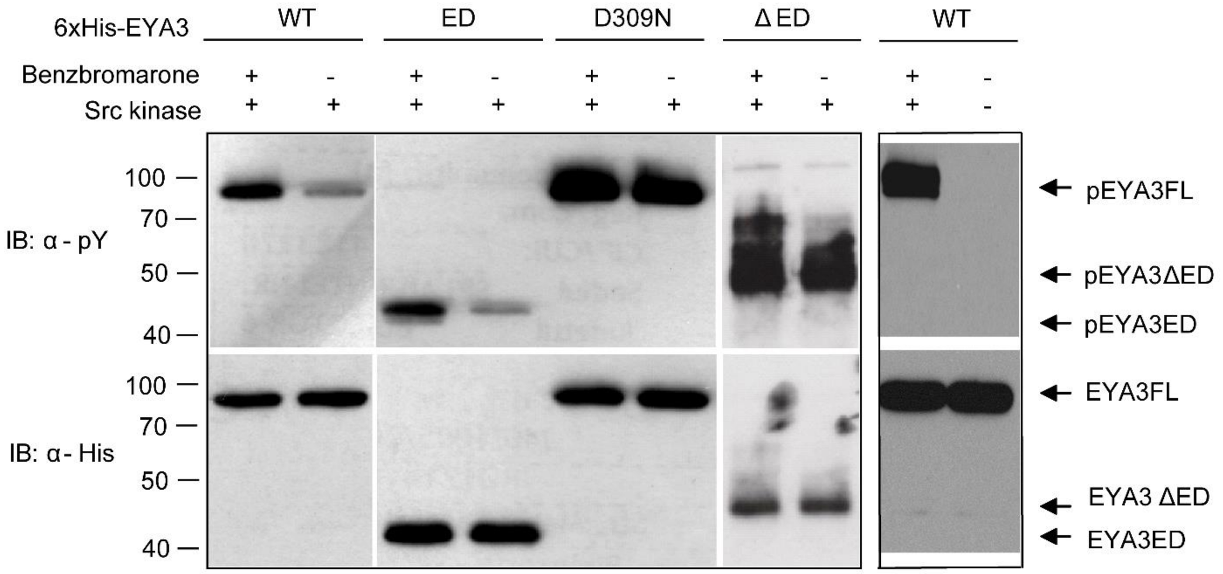

Supplementary Figure S4. Phosphorylation of His-tagged EYA3 by Src kinase and its autodephosphorylation. EYA3 (WT, D309N mutant, N-terminal and C-terminal domain) proteins were incubated with active GST-Src kinase (SignalChem) in kinase buffer assay. In parallel, samples were treated with 100 μM benzbromarone, EYA specific inhibitor**.** The phosphorylation level was analyzed by western blotting with anti-pTyr antibody.

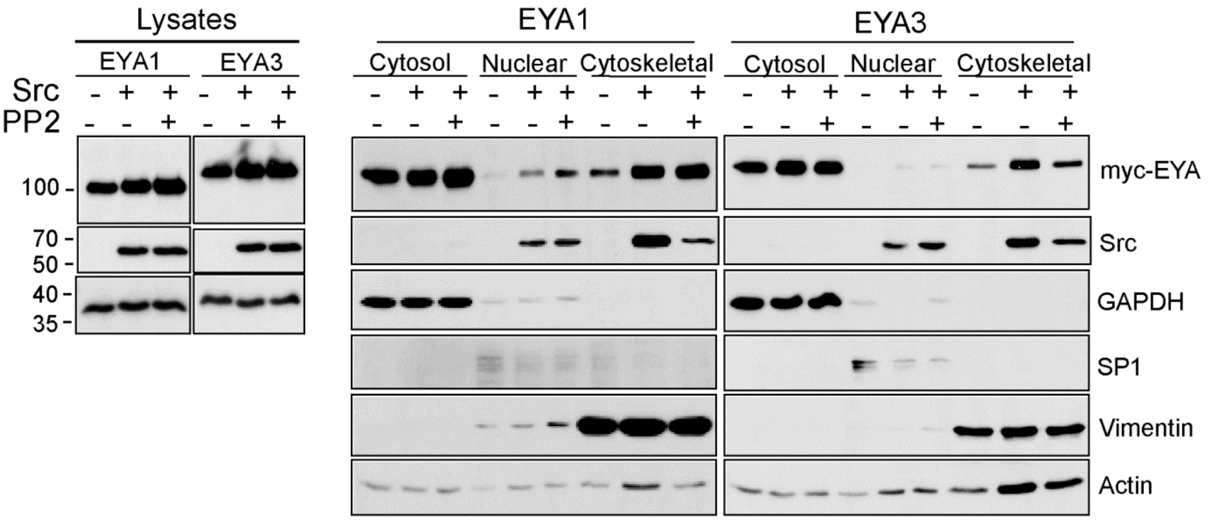

Supplementary Figure S5. EYA3 subcellular localization is dependent by Src kinase. EYA1 and EYA3 were overexpressed in 293T cells w/o Src kinase. The cells overexpressing both EYA and Src kinase were treated 4h post-transfection with either DMSO or PP2 (Src inhibitor) and analysed after 20h. The subcellular fractions were analyzed by immunoblot with specific markers: GAPDH (cytoplasm), calnexin (membrane), SP1 (nuclear), vimentin (cytoskeleton) and actin. The images are cropped from original blots.

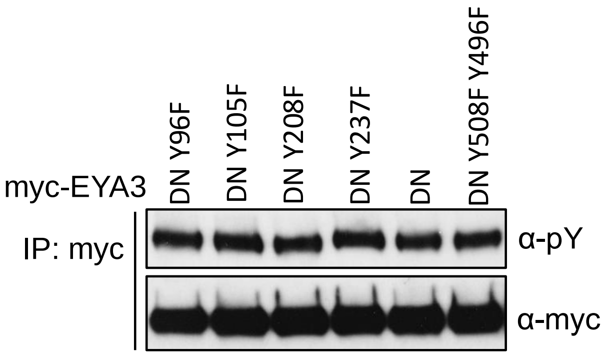

Supplementary Figure S6. The N-terminal single tyrosine mutations do not affect EYA3 phosphorylation. 293T cells were co-transfected with myc-tagged EYA3 constructs: D309N Y96F, D309N Y105F, D309N Y208F, D309N Y237F, D309N, D309N Y496F Y508F and Src kinase. Myc-tagged EYA proteins were immunoprecipitated using myc antibody and the the phosphorylation signal was analyzed by western blotting with anti-pTyr antibody. The images are cropped from original blots**.**

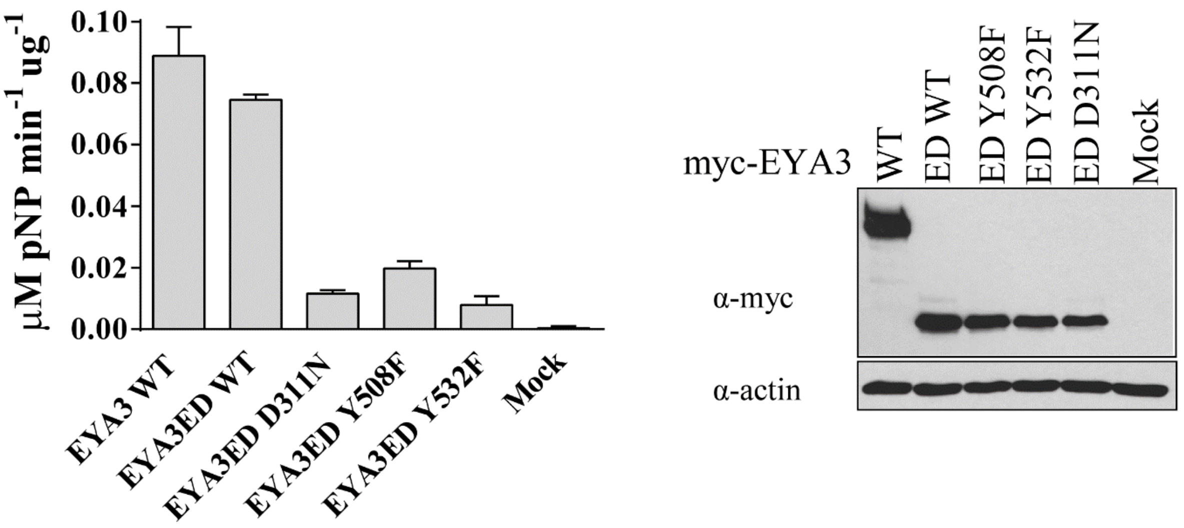

Supplementary Figure S7. The catalytic activity of EYA3ED is inactivated by single point mutations, Y508F and Y532F. Left panel: The histogram is showing the amount of product *(*pNP) formed in the phosphatase reaction by immunoprecipitated myc-EYA3ED proteins when pNPP was used as substrate. Error bars represent the standard deviation from two independent readings. Right panel: lysates showing the myc-tagged EYA3ED proteins used for immunoprecipitation. Note: myc-tagged EYA3 WT was used as a positive control.

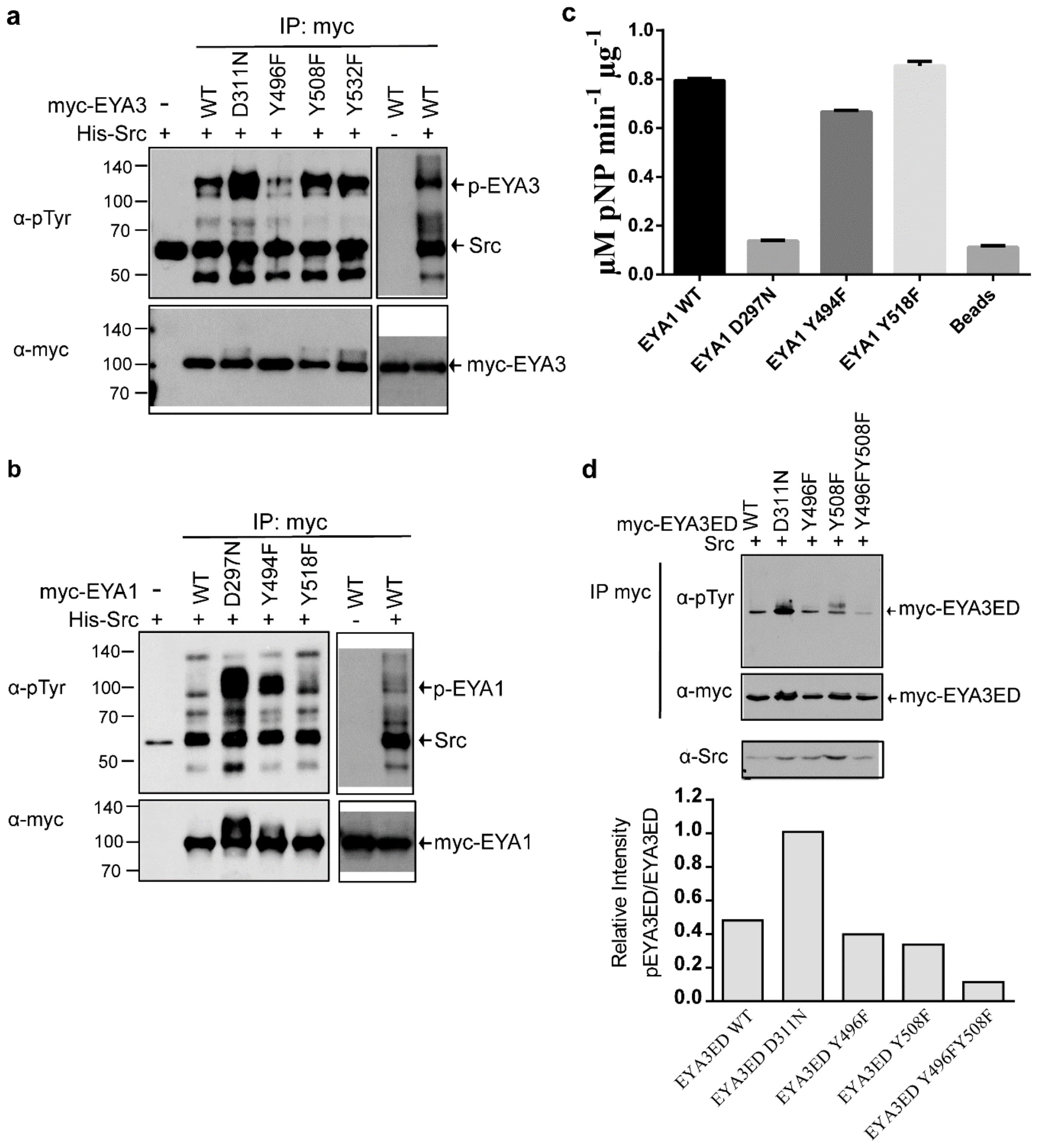

Supplementary Figure S8. Myc-tagged EYA1 and EYA3 proteins are phosphorylated by Src kinase. (a, b) EYA1 and EYA3 proteins were immunoprecipitated from 293T cells using anti-myc antibody and further incubated w/o active His-Src kinase (Millipore). The phosphorylation level of proteins was analysed by western blot with the indicated antibodies. **(c)** The histogram is showing the amount of product (pNP) formed in the phosphatase reaction for immunoprecipitated myc-EYA1 proteins when pNPP was used as substrate. Error bars represent the standard deviation from two independent readings. **(d)** myc-tagged EYA3ED mutants were immunoprecipitated from 293T cells co-expressing Src kinase. The phosphorylation signal of proteins was analysed by western blot using the indicated antibodies. The ratio between relative phosphorylation levels of EYA3ED normalized to the total immunoprecipitated protein was represented in the histogram. The bands were quantified using ImageJ software.

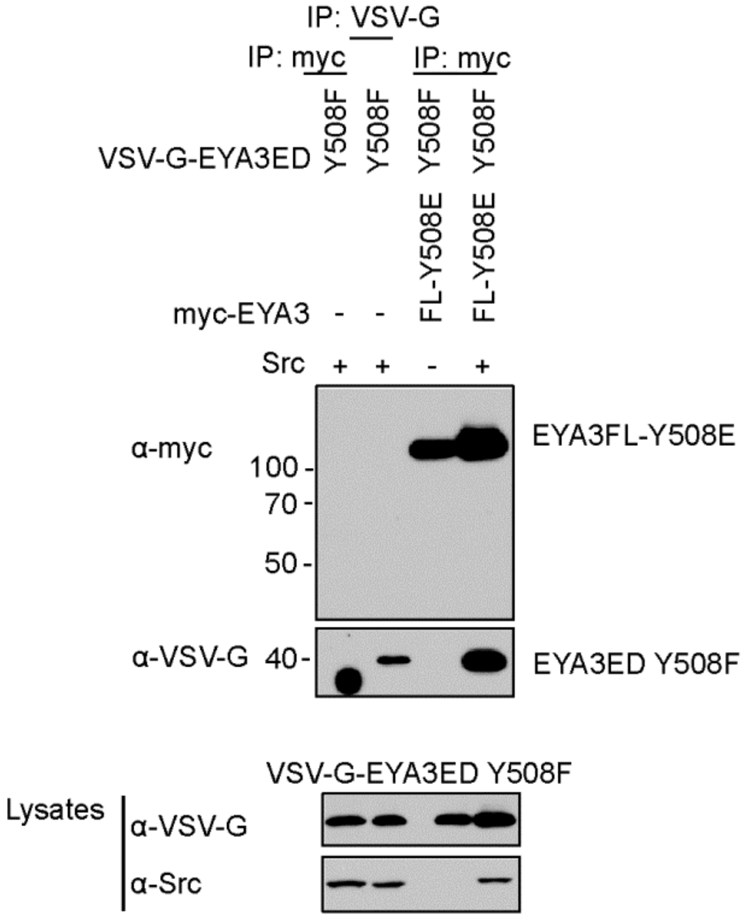

Supplementary Figure S9. EYA3-EYA3 interaction is dependent by Src phosphorylation. VSV-G-EYA3ED-Y508F and myc-EYA3FL-Y508E were cotransfected w/o Src kinase in 293T cells. The overexpressed myc tagged protein was immunoprecipitated with myc antibody and the VSV-G trapped protein was visualized by western blot using anti-VSV-G antibody. (&) VSV-G-EYA3ED-Y508F was cotransfected only with Src kinase and incubated with either Protein G beads and anti-myc antibody in order to eliminate the unspecific binding or with Protein G and anti-VSV-G antibody and used as positive control.

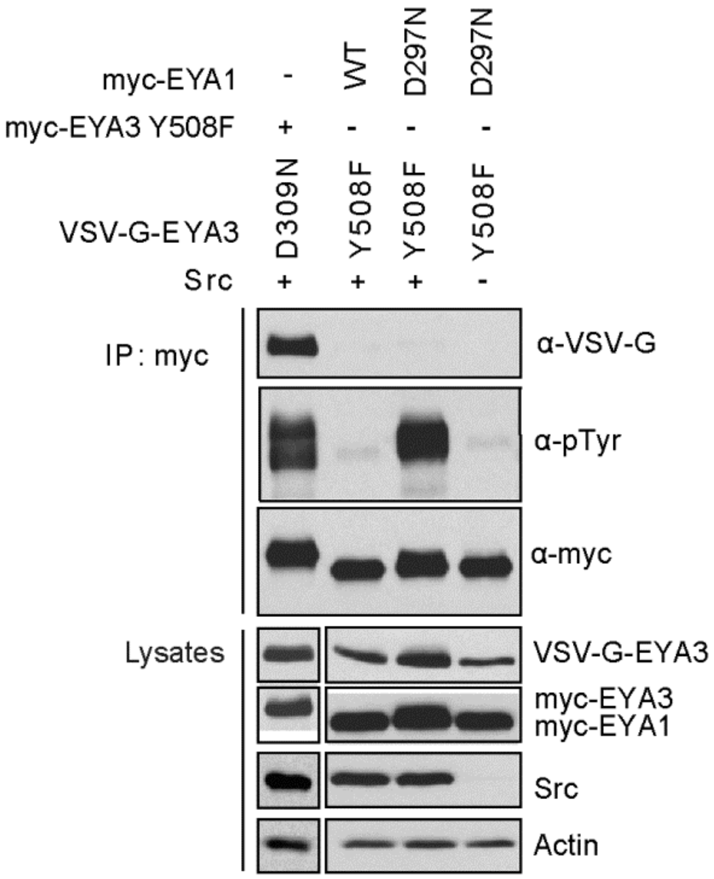

Supplementary Figure S10. EYA3 is not interacting with EYA1. Myc-tagged EYA1 WT and inactive mutant D297N were co-transfected with Src kinase and VSV-G-tagged EYA3 Y508F. Myc-EYA3 Y508F co-transfected with VSV-G-Eya3 D309N and Src kinase was used as positive control. Myc-tagged EYA proteins were immunoprecipitated using myc antibody and the co-immunoprecipitated protein was analyzed by western blotting with anti-VSV-G antibody. The images are cropped from original blots**.**

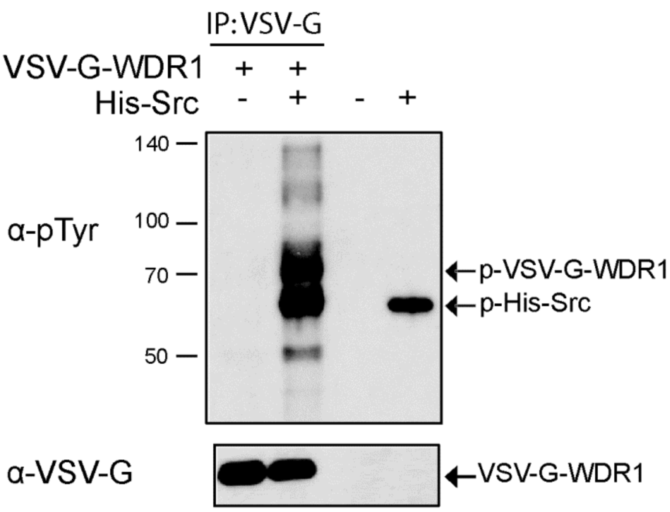

Supplementary Figure S11. WDR1 is phosphorylated by Src kinase *in vitro*. VSV-G-WDR1 protein was immunoprecipitated from 293T cells and further incubated w/o active His-Src kinase (Millipore) for 1h at 30°C in kinase buffer assay.

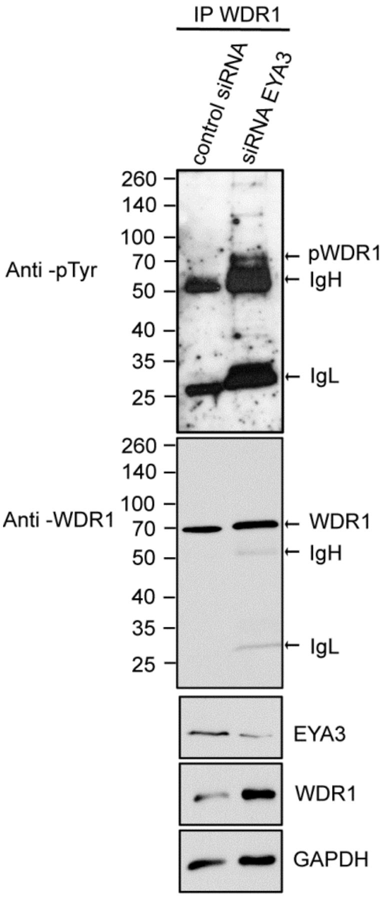

Supplementary Figure S12 Tyrosine phosphorylation of WDR1 is increased in cells treated with siRNA EYA3. WDR1 protein was immunoprecipitated from MCF7 cells treated for 24h with siRNA control or siRNA EYA3. Subsequently, WDR1 was analysed by western blot using anti-pTyr and anti-WDR1 antibodies.

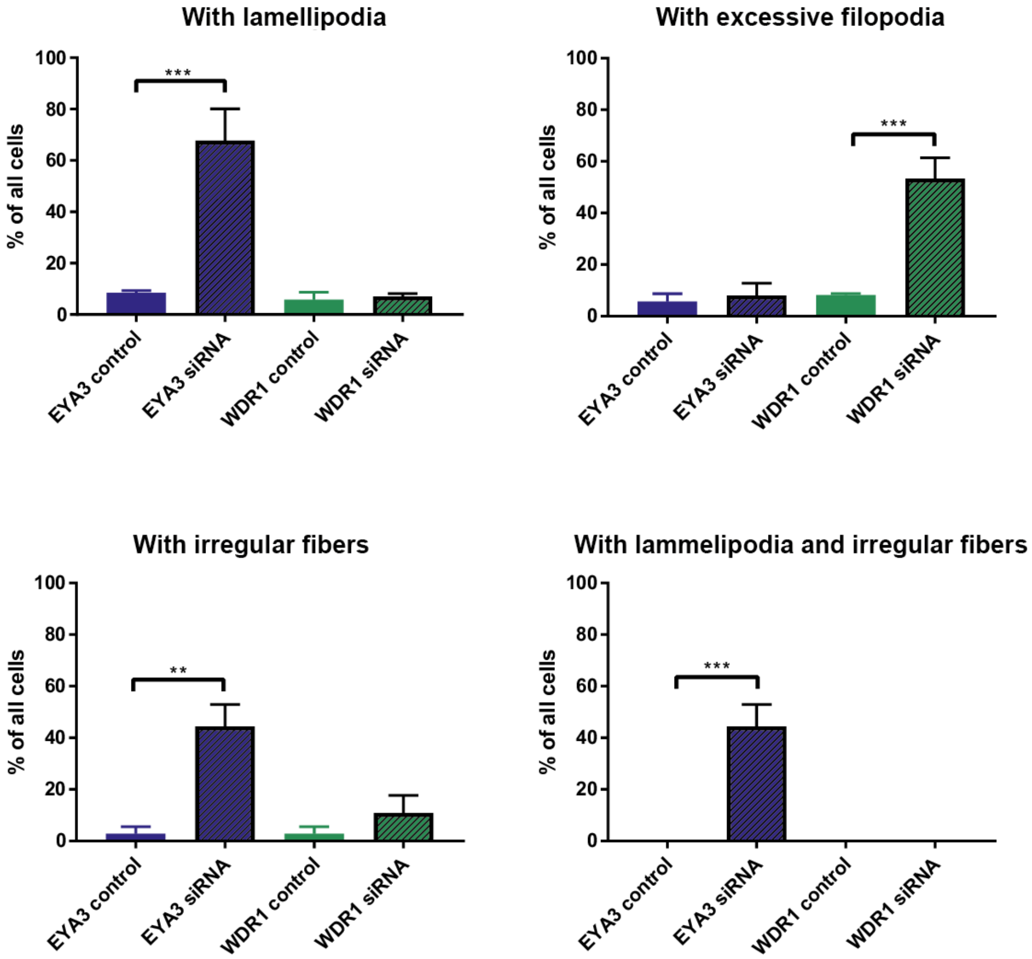

Supplementary Figure S13. EYA3 and WDR1 are involved in actin cytoskeleton rearrangement. Both proteins were knocked down in MCF7 cells and the actin cytoskeleton was analyzed as in Figure 7. Knockdown of EYA3 markedly increased the prevalence of lammelipodia and irregularly shaped actin fibers. WDR1 knockdown lead to an increase in cells having excessive amounts of filopodia. The bars show means ± SEM from three independent experiments. Significance was tested using one-way ANOVA, followed by Bonferronis correction.

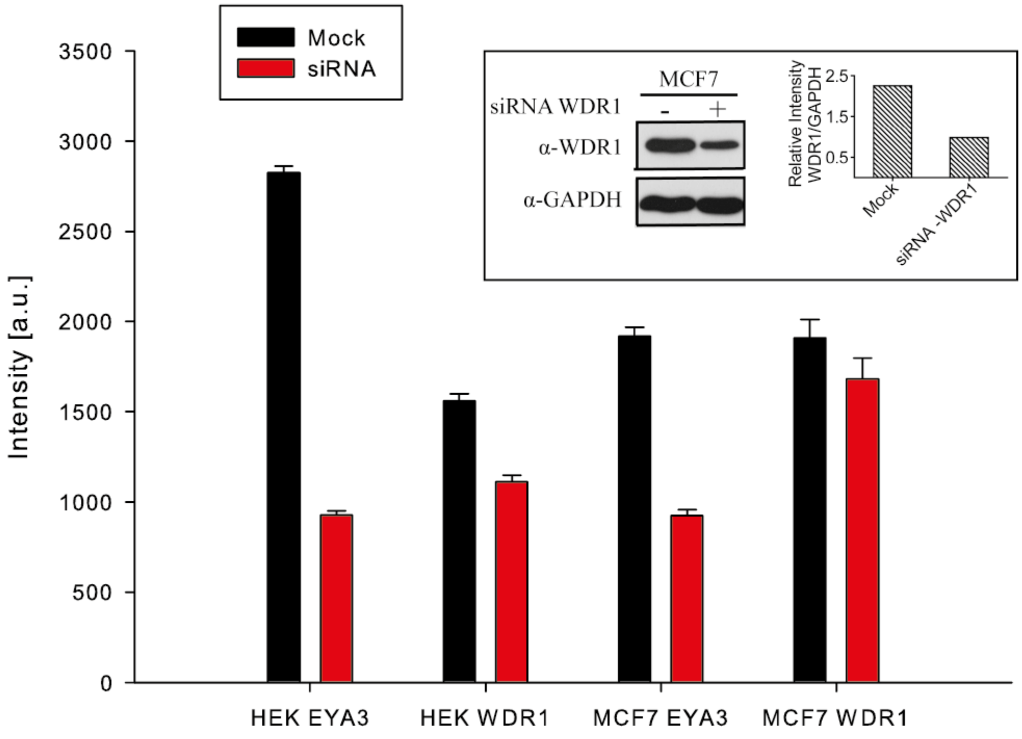

Supplementary Figure S14. KD efficiency of the siRNA employed in this study, as assessed by immunostainings and immunoblots. HEK and MCF7 cells were transfected with siRNA against WDR1, EYA3 or respective control. Each KD solution consisted of a mix of three different siRNA specific to the target protein. Cells were then stained against the respective proteins and the average intensity of the staining as compared. We tested all conditions by fluorescence, and all constructs appeared to function, although the difference was small in MCF7 cells for WDR1. To test this futher, we analyzed these cells by Western Blotting, which confirmed that the difference was indeed measurable, both in MCF7 and HEK cells (see inset). The bars show means ± SEM from three independent experiments.

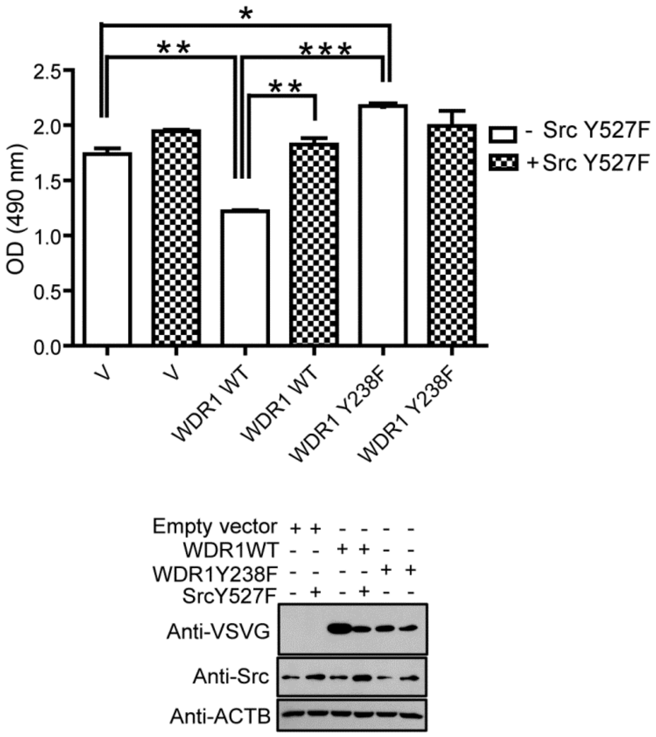

Supplementary Figure S15. WDR1 overexpression decreases the proliferation of MCF7 cells. The upper graphic shows the comparison of cell viability between cells overexpressing either WDR1 WT or WDR1Y238F with or without Src kinase (* $p < 0.05$; ** $p < 0.01$; *** $p < 0.001$). Significance was tested using unpaired t-test (GraphPad Prism 6). The lower panel shows both the WDR1 and Src protein levels at 24 h post transfection by western blot using the indicated antibodies.

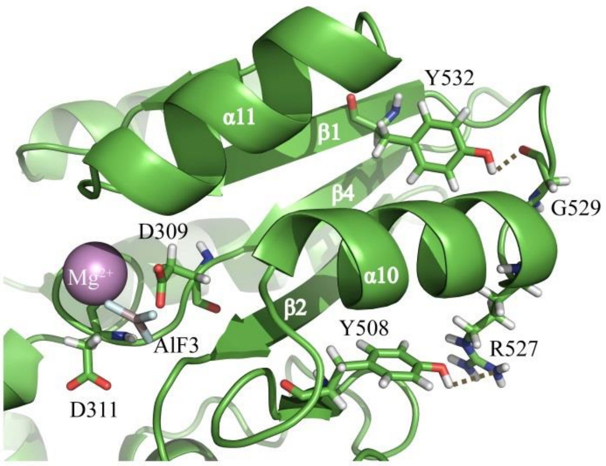

Supplementary Figure S16. Model of hEYA3 structure predicting consequences of Y508F and Y532F point mutations. Y508 and Y532 form hydrogen bonds with nitrogen atoms of R527 and oxygen atom of G529 peptide bond, respectively. Mutation of either Y508 or Y532 to F should abrogate mentioned interactions inducing perturbations of active site architecture. Active site is defined by D309, D311, Mg^{2+} and AlF_3 mimicking phosphate moiety of phospho-aspartyl enzyme intermediate. AlF3 has been positioned into the model by superposition to EYA3 model structure with structure of EYA2-AlF_3 complex (Protein Data Bank code: 3HB1).

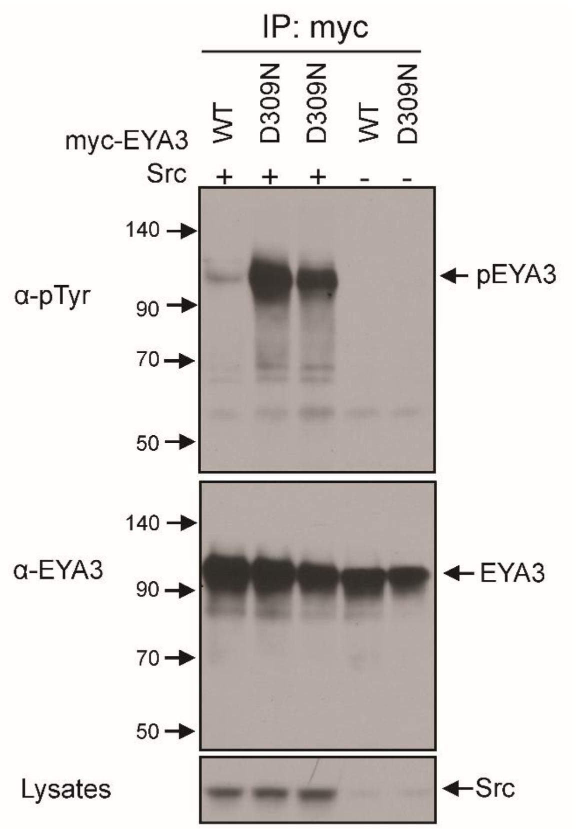

Supplementary Figure S17. Uncropped blots for Figure 1a. EYA3 proteins is phosphorylated by Src kinase and can autodephosphorylate. Wild-type EYA3 and its catalytically inactive mutant were co-expressed in 293T cells with Src kinase. The proteins were immunoprecipitated from cell lysates with anti-myc antibody and analyzed by western blotting with anti-pTyr and anti-myc antibodies. Whole cell lysates (WCLs) were immunoblotted with anti-Src antibody.

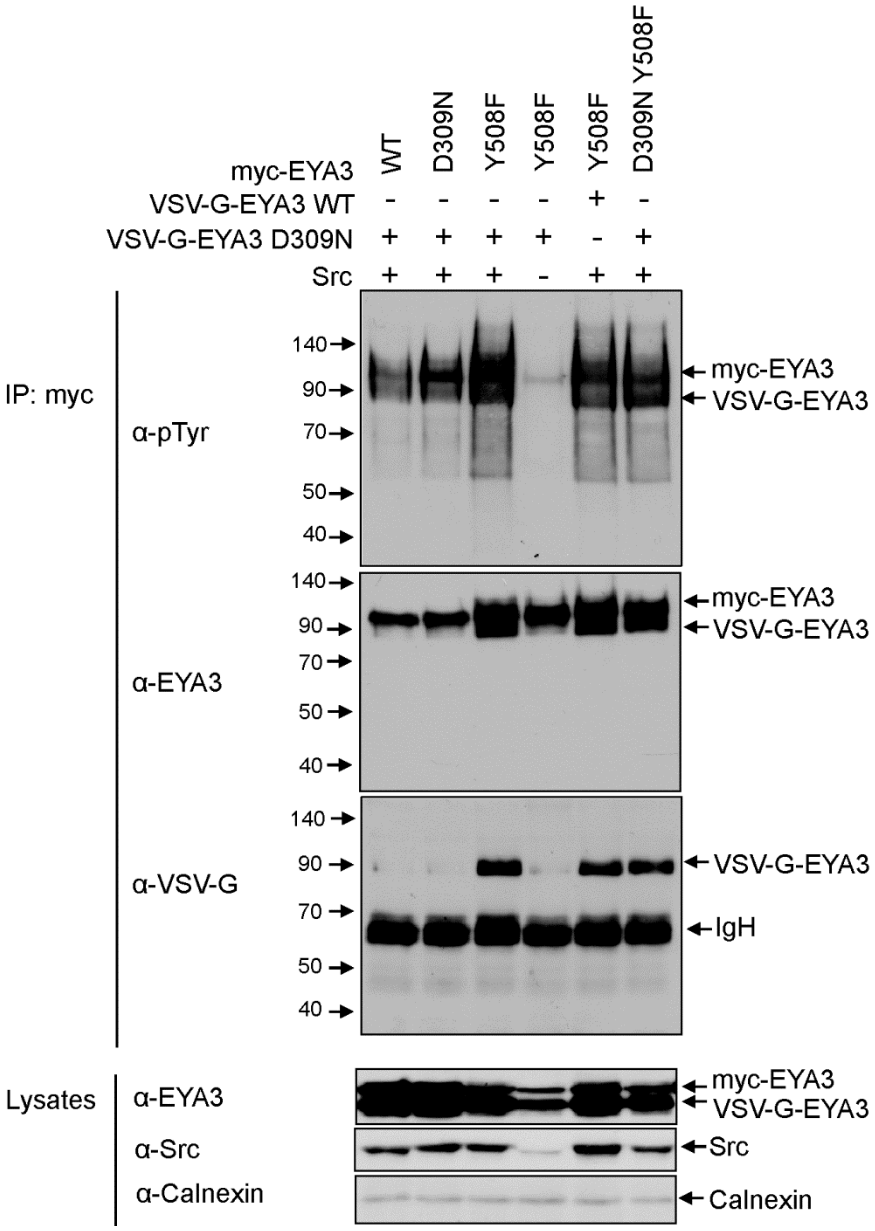

Supplementary Figure S18. Uncropped blots for Figure 3b. EYA3 Y508F and EYA3 Y532F proteins are new, specific trapping mutants for EYA3 phosphatase. Myc-tagged EYA3 (WT, D309N, Y508F and D309N Y508F) and VSV-G-EYA3 (WT and D309N) were coexpressed in 293T cell w/o Src kinase. Myc-tagged EYA proteins were immunoprecipitated from cell lysates and trapped proteins were analyzed by western blotting with indicated antibodies. WCLs were immunoblotted with anti-Src and anti-calnexin.

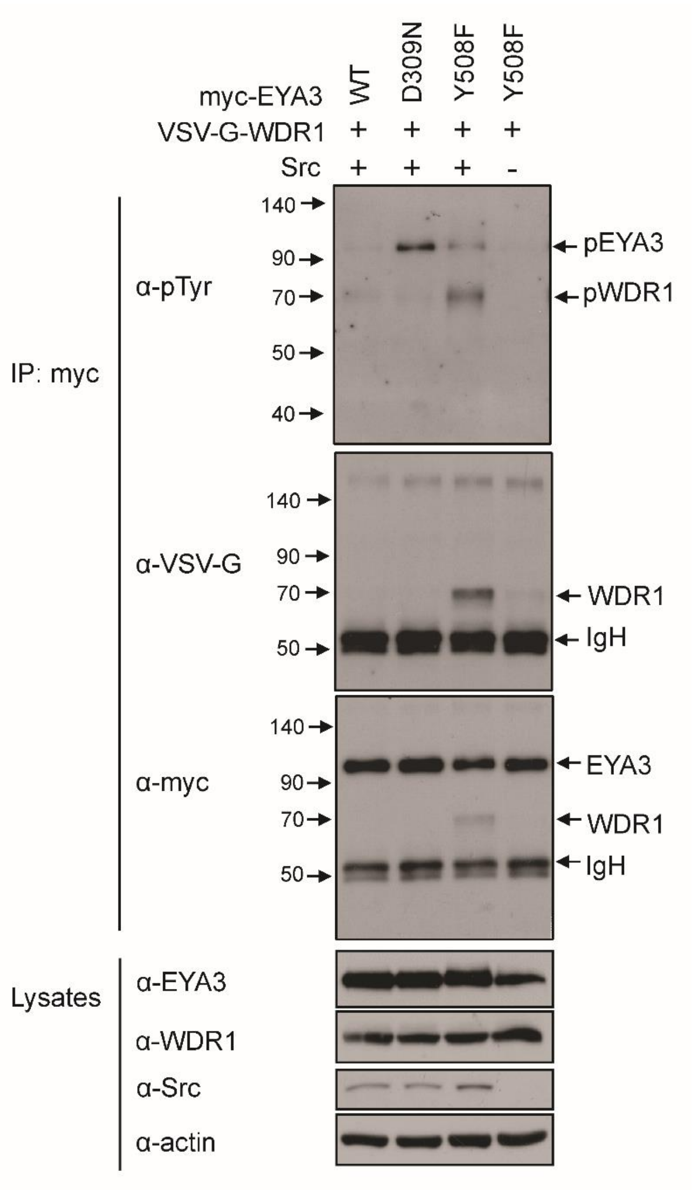

Supplementary Figure S19. Uncropped blots for Figure 6a. VSV-G-WDR1 WT was co-expressed in 293T cells w/o Src kinase and different myc-tagged EYA3 constructs: EYA3 WT, D309N and Y508F. Myc-tagged proteins were immunoprecipitated, followed by western blot analysis with anti-pTyr, anti-VSV-G and anti-myc antibodies. WCLs were analyzed with antibodies against EYA3, WDR1, Src and actin. Note: the western blot membrane used for dectection with anti-VSV-G was reused for detection with anti-myc, without stripping.

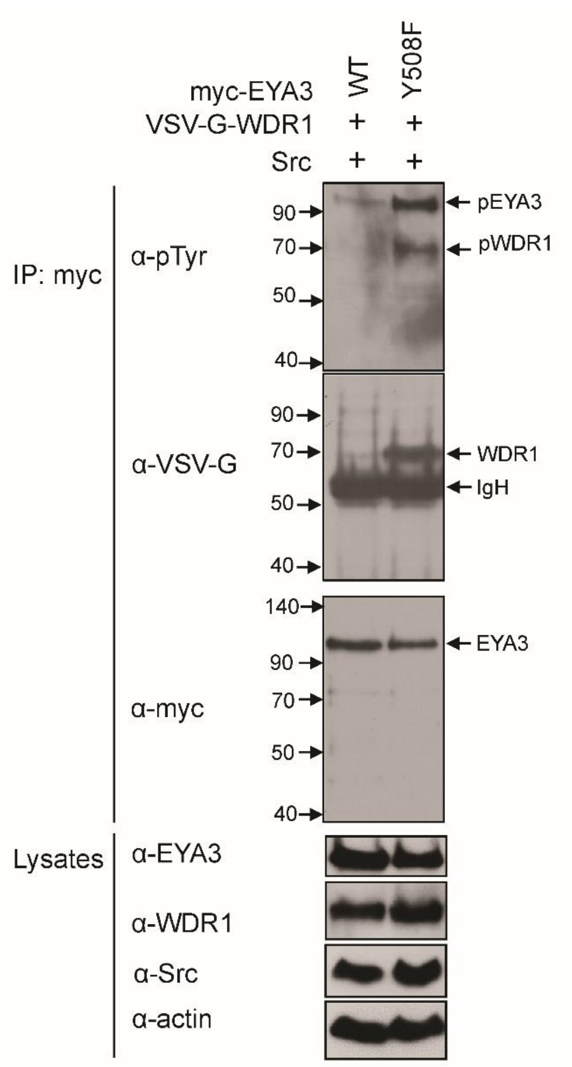

Supplementary Figure S20. Uncropped blots for Figure 6c. MCF7 cells were transfected with either myc-EYA3 WT or Y508F and VSV-G-WDR1WT and Src kinase. Myc-tagged proteins were immunoprecipitated, followed by western blot analysis with anti-pTyr, anti-VSV-G and anti-myc antibodies. WCLs were analyzed with antibodies against EYA3, WDR1, Src and actin (bottom).

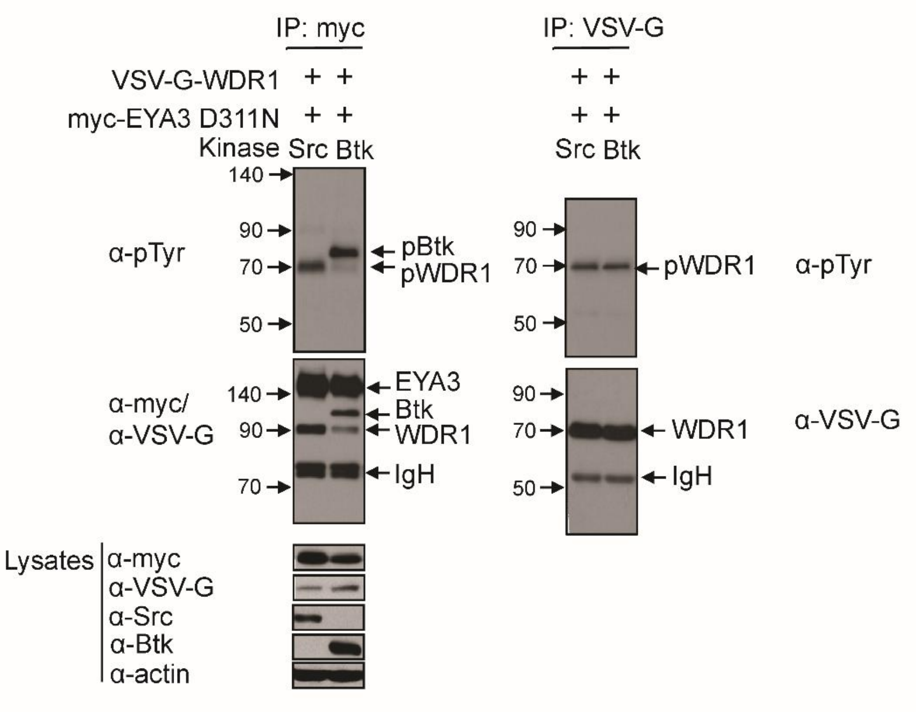

Supplementary Figure S21. Uncropped blots for Figure 6e. Myc-EYA3 D311N and VSV-G-WDR1 were transfected either with Src or Btk kinase in 293T cells. Myc-tagged proteins were immunoprecipitated, followed by western blot analysis with anti-pTyr, anti-VSV-G and anti-myc antibodies. VSV-G tagged proteins were immunoprecipitated, followed by western blot analysis with anti-pTyr and anti-VSV-G. WCLs were analyzed with antibodies against EYA3, WDR1, Src and actin. Note: the western blot membrane used for detection with anti-myc (left panel) was reprobed with anti-VSV-G antibody without stripping the membrane. Myc-tagged Btk was used in experiment.

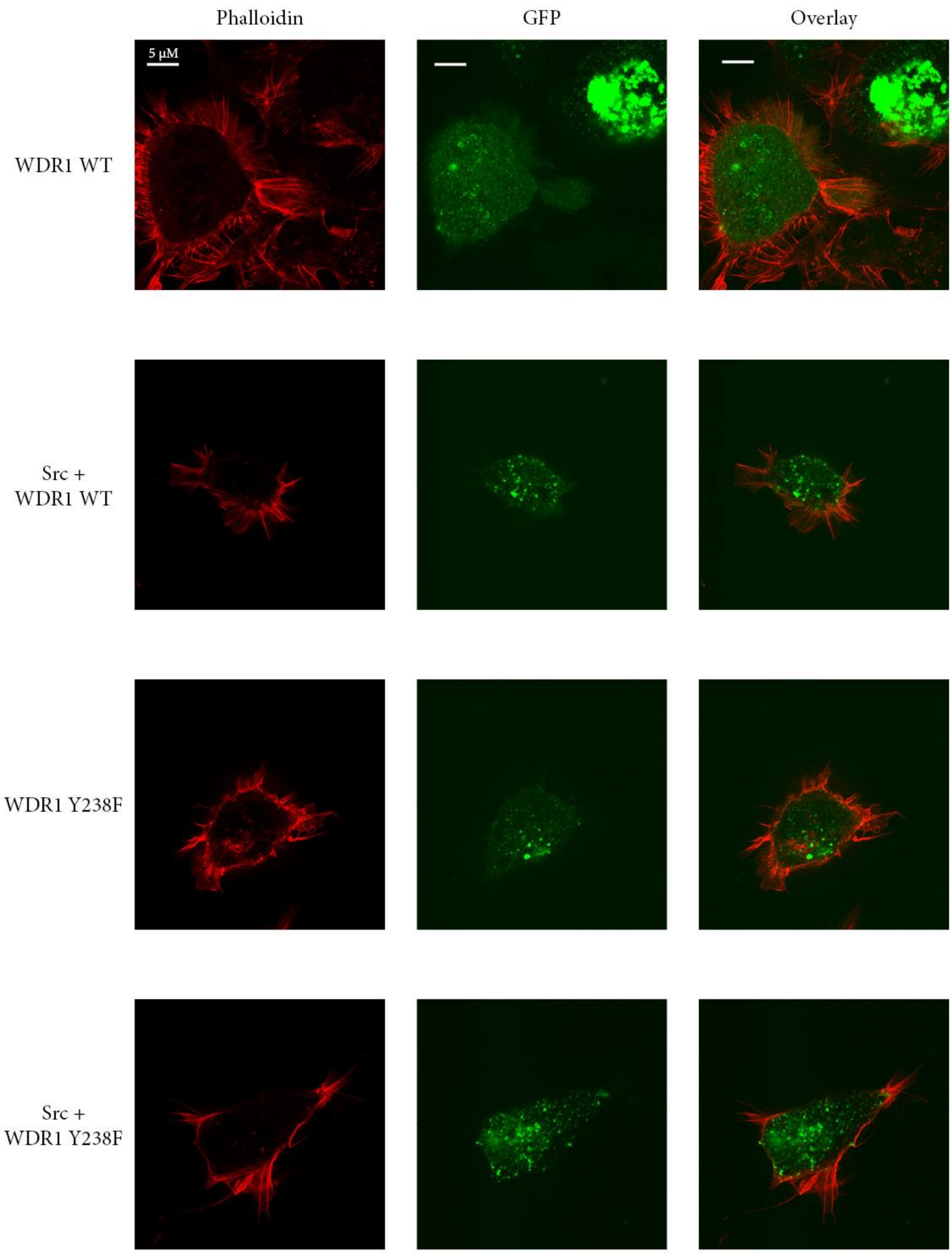

Supplementary Figure S22. HEK293T cells showing both GFP-WDR1 fluorescence and actin phalloidin stain. HEK cells were either transfected with WDR1/WDR1 Y238F alone, or co-transfected with Src kinase. Only cells showing both GFP transfection and phalloidin stain of actin filaments were analysed in Fig 7.

Supplementary Table 1. EYA1 and EYA3 mutants

EYA1				EYA3			
Mutants	Activity on pNPP	Trapping mutant	Autodephosphorylation capacity	Mutants	Activity on pNPP	Trapping mutants	Autodephosphorylation capacity
EYA1 WT	+	–	+	EYA3 WT	+	–	+
D295N	–	–	–	D309N	–	–	–
D297N	–	+	–	D311N	–	+	–
Y482F	Nd	Nd	Nd	Y496F	+	Nd	+
Y494F	+	Nd	+[b]	Y508F	–	+	–
Y518F	+	Nd	+	Y532F	–	+	–
D297N Y482F	–[a]	Nd	Nd	D309N Y496F	–	Nd	–
D297N Y494F	–[a]	Nd	–	D309N Y508F	–	+	–
D297N Y518F	–[a]	Nd	–	D309N Y532F	–	Nd	–
D297N Y482F Y494F	–[a]	Nd	Nd	D309N Y496F Y508F	–[c]	Nd	–
D297N Y482F Y494F Y518F	–[a]	Nd	Nd	D309N Y496F Y508F Y532F	–[c]	Nd	–
				D309N Y96F	–[c]	Nd	–
				D309N Y105F	–[c]	Nd	–
				D309N Y208F	–[c]	Nd	–
				D309N Y237F	–[c]	Nd	–

Notes:
Nd - Not determined
a - By similarity with D297N inactive mutant
b – Autodephosphorylation capacity is lower compared with EYA1 wild-type, when Src kinase was used for phosphorylation assay *in vitro*
c - By similarity with D309N inactive mutant

Supplementary Table 2. Total number of cells analysed by STED microscopy

Conditions	Total number of cells
**Figure 7**	
Src	49
Src WDR-mut	42
Src WDR-wt	52
Untransfected	52
Wdr-mut	51
WDR wt	52
**Supplementary Figure S13**	
EYA3 ctrl	28
EYA3 siRNA	30
WDR1 ctrl	26
WDR1 siRNA	31

Supplementary Table 3. One way ANOVA results for Category Lammelipodia.

Bonferroni's multiple comparisons test	Mean Diff.	95.00% CI of diff.	Significant?	Summary	Adjusted P Value
Untransfected vs. Src	-27.71	-86.3 to 30.89	No	ns	>0.9999
Untransfected vs. WDR1 WT	-47.12	-105.7 to 11.48	No	ns	0.1876
Untransfected vs. WDR1 Y238F	-62.88	-121.5 to -4.283	Yes	*	0.0308
Untransfected vs. WDR1 WT + Src	-49.23	-107.8 to 9.372	No	ns	0.1470
Untransfected vs. WDR1 Y238F + Src	-49.78	-108.4 to 8.816	No	ns	0.1379
Src vs. WDR1 WT	-19.41	-78.01 to 39.18	No	ns	>0.9999
Src vs. WDR1 Y238F	-35.17	-93.77 to 23.42	No	ns	0.7348
Src vs. WDR1 WT + Src	-21.52	-80.12 to 37.08	No	ns	>0.9999
Src vs. WDR1 Y238F + Src	-22.07	-80.67 to 36.52	No	ns	>0.9999
WDR1 WT vs. WDR1 Y238F	-15.76	-74.36 to 42.84	No	ns	>0.9999
WDR1 WT vs. WDR1 WT + Src	-2.105	-60.7 to 56.49	No	ns	>0.9999
WDR1 WT vs. WDR1 Y238F + Src	-2.661	-61.26 to 55.94	No	ns	>0.9999
WDR1 Y238F vs. WDR1 WT + Src	13.65	-44.94 to 72.25	No	ns	>0.9999
WDR1 Y238F vs. WDR1 Y238F + Src	13.1	-45.5 to 71.7	No	ns	>0.9999
WDR1 WT + Src vs. WDR1 Y238F + Src	-0.5553	-59.15 to 58.04	No	ns	>0.9999

Supplementary Table 4. One way ANOVA results for Category Excessive filopodia.

Bonferroni's multiple comparisons test	Mean Diff.	95.00% CI of diff.	Significant?	Summary	Adjusted P Value
Untransfected vs. Src	-51.41	-91.05 to -11.78	Yes	**	0.0073
Untransfected vs. WDR1 WT	-14.71	-54.34 to 24.93	No	ns	>0.9999
Untransfected vs. WDR1 Y238F	-3.081	-42.71 to 36.55	No	ns	>0.9999
Untransfected vs. WDR1 WT + Src	-23.08	-62.71 to 16.55	No	ns	0.8256
Untransfected vs. WDR1 Y238F + Src	-19.56	-59.19 to 20.07	No	ns	>0.9999
Src vs. WDR1 WT	36.71	-2.925 to 76.34	No	ns	0.0821
Src vs. WDR1 Y238F	48.33	8.701 to 87.97	Yes	*	0.0119
Src vs. WDR1 WT + Src	28.33	-11.3 to 67.97	No	ns	0.3429
Src vs. WDR1 Y238F + Src	31.85	-7.781 to 71.48	No	ns	0.1882
WDR1 WT vs. WDR1 Y238F	11.63	-28.01 to 51.26	No	ns	>0.9999
WDR1 WT vs. WDR1 WT + Src	-8.374	-48.01 to 31.26	No	ns	>0.9999
WDR1 WT vs. WDR1 Y238F + Src	-4.856	-44.49 to 34.78	No	ns	>0.9999
WDR1 Y238F vs. WDR1 WT + Src	-20	-59.63 to 19.63	No	ns	>0.9999
WDR1 Y238F vs. WDR1 Y238F + Src	-16.48	-56.11 to 23.15	No	ns	>0.9999
WDR1 WT + Src vs. WDR1 Y238F + Src	3.518	-36.11 to 43.15	No	ns	>0.9999

Supplementary Table 5. One way ANOVA results for Category Irregular fibres.

Bonferroni's multiple comparisons test	Mean Diff.	95.00% CI of diff.	Significant?	Summary	Adjusted P Value
Untransfected vs. Src	0	-26.46 to 26.46	No	ns	>0.9999
Untransfected vs. WDR1 WT	-1.852	-28.31 to 24.6	No	ns	>0.9999
Untransfected vs. WDR1 Y238F	-70.78	-97.24 to -44.33	Yes	****	<0.0001
Untransfected vs. WDR1 WT + Src	-8.519	-34.97 to 17.94	No	ns	>0.9999
Untransfected vs. WDR1 Y238F + Src	-33.33	-59.79 to -6.878	Yes	**	0.0092
Src vs. WDR1 WT	-1.852	-28.31 to 24.6	No	ns	>0.9999
Src vs. WDR1 Y238F	-70.78	-97.24 to -44.33	Yes	****	<0.0001
Src vs. WDR1 WT + Src	-8.519	-34.97 to 17.94	No	ns	>0.9999
Src vs. WDR1 Y238F + Src	-33.33	-59.79 to -6.878	Yes	**	0.0092
WDR1 WT vs. WDR1 Y238F	-68.93	-95.39 to -42.48	Yes	****	<0.0001
WDR1 WT vs. WDR1 WT + Src	-6.667	-33.12 to 19.79	No	ns	>0.9999
WDR1 WT vs. WDR1 Y238F + Src	-31.48	-57.94 to -5.026	Yes	*	0.0144
WDR1 Y238F vs. WDR1 WT + Src	62.27	35.81 to 88.72	Yes	****	<0.0001
WDR1 Y238F vs. WDR1 Y238F + Src	37.45	11 to 63.91	Yes	**	0.0035
WDR1 WT + Src vs. WDR1 Y238F + Src	-24.81	-51.27 to 1.641	No	ns	0.0758

Supplementary Table 6. One way ANOVA results for Category Lammelipodia and irregular fibres.

Bonferroni's multiple comparisons test	Mean Diff.	95.00% CI of diff.	Significant?	Summary	Adjusted P Value
Untransfected vs. Src	0	-28.03 to 28.03	No	ns	>0.9999
Untransfected vs. WDR1 WT	-1.852	-29.88 to 26.17	No	ns	>0.9999
Untransfected vs. WDR1 Y238F	-69.4	-97.42 to -41.37	Yes	****	<0.0001
Untransfected vs. WDR1 WT + Src	-1.852	-29.88 to 26.17	No	ns	>0.9999
Untransfected vs. WDR1 Y238F + Src	-27.78	-55.8 to 0.248	No	ns	0.0530
Src vs. WDR1 WT	-1.852	-29.88 to 26.17	No	ns	>0.9999
Src vs. WDR1 Y238F	-69.4	-97.42 to -41.37	Yes	****	<0.0001
Src vs. WDR1 WT + Src	-1.852	-29.88 to 26.17	No	ns	>0.9999
Src vs. WDR1 Y238F + Src	-27.78	-55.8 to 0.248	No	ns	0.0530
WDR1 WT vs. WDR1 Y238F	-67.54	-95.57 to -39.52	Yes	****	<0.0001
WDR1 WT vs. WDR1 WT + Src	0	-28.03 to 28.03	No	ns	>0.9999
WDR1 WT vs. WDR1 Y238F + Src	-25.93	-53.95 to 2.1	No	ns	0.0827
WDR1 Y238F vs. WDR1 WT + Src	67.54	39.52 to 95.57	Yes	****	<0.0001
WDR1 Y238F vs. WDR1 Y238F + Src	41.62	13.59 to 69.64	Yes	**	0.0023
WDR1 WT + Src vs. WDR1 Y238F + Src	-25.93	-53.95 to 2.1	No	ns	0.0827
